# Dependence of Micelle Size and Shape on Detergent Alkyl Chain Length and Head Group

**DOI:** 10.1371/journal.pone.0062488

**Published:** 2013-05-08

**Authors:** Ryan C. Oliver, Jan Lipfert, Daniel A. Fox, Ryan H. Lo, Sebastian Doniach, Linda Columbus

**Affiliations:** 1 Department of Chemistry, University of Virginia, Charlottesville, Virginia, United States of America; 2 Department of Bionanoscience, Kavli Institute of Nanoscience, Delft University of Technology, Delft, The Netherlands; 3 Departments of Physics and Applied Physics, Biophysics Program, Stanford, California, United States of America; 4 Stanford Synchrotron Radiation Laboratory, Stanford University, Stanford, California, United States of America; Dalhousie University, Canada

## Abstract

Micelle-forming detergents provide an amphipathic environment that can mimic lipid bilayers and are important tools for solubilizing membrane proteins for functional and structural investigations *in vitro*. However, the formation of a soluble protein-detergent complex (PDC) currently relies on empirical screening of detergents, and a stable and functional PDC is often not obtained. To provide a foundation for systematic comparisons between the properties of the detergent micelle and the resulting PDC, a comprehensive set of detergents commonly used for membrane protein studies are systematically investigated. Using small-angle X-ray scattering (SAXS), micelle shapes and sizes are determined for phosphocholines with 10, 12, and 14 alkyl carbons, glucosides with 8, 9, and 10 alkyl carbons, maltosides with 8, 10, and 12 alkyl carbons, and lysophosphatidyl glycerols with 14 and 16 alkyl carbons. The SAXS profiles are well described by two-component ellipsoid models, with an electron rich outer shell corresponding to the detergent head groups and a less electron dense hydrophobic core composed of the alkyl chains. The minor axis of the elliptical micelle core from these models is constrained by the length of the alkyl chain, and increases by 1.2–1.5 Å per carbon addition to the alkyl chain. The major elliptical axis also increases with chain length; however, the ellipticity remains approximately constant for each detergent series. In addition, the aggregation number of these detergents increases by ∼16 monomers per micelle for each alkyl carbon added. The data provide a comprehensive view of the determinants of micelle shape and size and provide a baseline for correlating micelle properties with protein-detergent interactions.

## Introduction

Detergents have played a significant role in advancing the understanding of membrane protein structure and function. Detergent monomers in solution self-assemble at and above the critical micelle concentration (cmc) to form micelles. Micelles adopt globular shapes (e.g., spheres, ellipsoids, and cylinders) of various sizes, determined by the detergent head group structure and alkyl chain length [1]. The hydrophilic head groups of the detergent comprise a solvent-exposed outer shell, while the hydrophobic alkyl chains are sequestered from water and form the micelle core. Thus, the cross-sectional composition of the micelle is structurally similar to the lipid bilayer with hydrocarbon chains bound by hydrophilic head groups.

Consequently, micelles are often employed as mimics of lipid bilayers to solubilize and stabilize integral membrane proteins for structural and functional *in vitro* studies [2]–[5]. Although nonmicellar amphiphilic systems, such as nanodiscs [6] and bicelles [7], have been used in membrane protein studies, so far detergents have demonstrated more successes in high-resolution structure determination of membrane proteins. Nonetheless, stable, functional protein-detergent complexes (PDC) are difficult to obtain because protein denaturation and aggregation often occur. Determining the optimal conditions that yield a properly folded membrane protein relies heavily on exhaustive screening of detergents [8]–[11]. This need for empirical detergent screening stems from a lack of understanding of the physical forces between the detergent micelle and membrane protein.

Micelle size, shape, and detergent concentration need to be considered in evaluating a PDC for structural and biochemical studies. For instance, recent evidence suggests that the micelle hydrophobic thickness needs to match that of the membrane protein to maintain proper fold and function [12]–[14] (similar to the hydrophobic match proposed for membrane proteins in bilayers [15]–[19]). Therefore, a systematic investigation of the size and shape determinants of pure micelles will provide a baseline and foundation for the further understanding of micelle-protein interactions.

In this study, the sizes and shapes of micelles formed by four classes of detergents, phosphocholines, maltosides, glucosides, and lysophosphatidyl glycerols (Table 1), are investigated using small-angle X-ray scattering (SAXS). The detergents were selected based on their prevalence in membrane protein structural biology. Approximately 40% of the ∼115 membrane protein structures determined by NMR were prepared in dodecyl phosphocholine (FC12) micelles while nearly 40% of the ∼1200 membrane protein structures determined by X-ray crystallography were in octyl glucoside (OG), decyl maltoside (DM), or dodecyl maltoside (DDM) micelles [20]. Lysophosphatidyl glycerols (LPGs) have also facilitated solution NMR investigations of membrane proteins [21]. For each detergent class, multiple alkyl chain lengths were studied to address structure similarities within the class as well as common trends among all classes.

**Table 1:** Physical properties of pure detergents.

Detergent (abbreviation)	FW (Da)	cmc (mM)	*V*_mon_ a (Å^3^)	*ρ*_det_ b (e/Å^3^)	*N*_lit._
*n*-decylphosphocholine (FC10)	323	11^c^	494	0.360	24^c^, 45–53 [22]
*n*-dodecylphosphocholine (FC12)	351	1.5^c^	548	0.354	54^c^, 60–80 [22]
*n*-tetradecylphosphocholine (FC14)	380	0.12^c^	602	0.348	108^c^
*n*-octyl-β-d-glucopyranoside (OG)	292	18–20 [38]	419	0.382	87 [39], 27–100 [38]
*n*-nonyl-β-d-glucopyranoside (NG)	306	6.5^c^	446	0.377	133^c^
*n*-decyl-β-d-glucopyranoside (DG)	320	2.2 [40]	472	0.373	200–400 [41]
*n*-octyl-β-d-maltopyranoside (OM)	454	19.5^c^	590	0.416	6^c^, 26 [42]
*n*-decyl-β-d-maltopyranoside (DM)	483	1.8 [43]	644	0.407	69^c^, 82–90 [22]
*n*-dodecyl-β-d-maltopyranoside (DDM)	511	0.17 [44]	698	0.398	132 [36], 78–149 [44], 135–145 [22], [45]
1-myristoyl-2-hydroxy-*sn*-glycero-3- phosphor-(1′-*rac*-glycerol) (LMPG)	478	0.16 [46]	639	0.404	55^d^
1-palmityl-2-hydroxy-*sn*-glycero-3- phosphor-(1′-*rac*-glycerol) (LPPG)	507	0.018 [46]	693	0.395	160–170 [22]

a Monomer volumes were calculated from published specific densities, using the Tanford formula (*V_tail_* = N*(24.7+26.9*n_c_*)) for alkyl chain volumes to adjust for different chain lengths.

b The detergent electron density values were computed by summing the number of electrons from the chemical composition and dividing by the molecular volume.

c Measurements performed by Anatrace (Affymetrix, Inc.). All cmcs are reported for conditions of detergent in H_2_O, except for the cmc of OM which is reported in 20 mM HEPES, pH 7.5 with 100 mM NaCl. Aggregation numbers from ref. [24] are reported in the same buffer used in this study.

d A measured value was not found in the literature; although many studies report this aggregation number. However, an estimate can be made from the PDC molecular weight reported by Tian *et al.* of ∼60 kD [47] (detergent contribution of 44 kD), which yields an aggregation number of ∼90.

To date, few experimental studies have systematically investigated the effects of detergent structure on micelle geometry. In addition, a limited number of detergents and alkyl chain lengths have been investigated and inconsistencies in buffer conditions and methodologies between studies complicate comparison of detergent micelle properties. This study experimentally correlates the monomer detergent structure with micelle physical properties so that micelle shapes and sizes can be more accurately predicted, in particular with a view towards assessing PDC structure and function.

The shapes and sizes of micelles formed by the eleven detergents (Table 1) were analyzed under the same experimental conditions. The model independent and dependent parameters were then analyzed to establish trends in micelle size and shape. With an increase in the alkyl chain by a carbon atom, the aggregation number increases by 16±3 detergent monomers. The short axis of the micelles increases with a distance expected for an additional alkyl carbon, while ellipticity (the ratio of the minor and major axes) is maintained. These results establish ellipsoid micelle models that are consistent through a detergent series and are expected to provide predictive measures for other detergent micelle size and shapes.

## Materials and Methods

### Sample Preparation

The detergents *n*-decyl-phosphocholine (FC10), *n*-dodecyl-phosphocholine (FC12), *n*-tetradecyl-phosphocholine (FC14), *n*-octyl-β-d-glucopyranoside (OG), *n*-nonyl-β-d-glucopyranoside (NG), *n*-decyl-β-d-glucopyranoside (DG), *n*-octyl-β-d-maltopyranoside (OM), *n*-decyl-β-d-maltopyranoside (DM), and *n*-dodecyl-β-d-maltopyranoside (DDM) (Fig. S1) were purchased from Anatrace (Affymetrix). The lysophospholipid detergents 1-myristoyl-2-hydroxy-*sn*-glycero-3-phospho-(1′-*rac*-glycerol) (14∶0 Lyso PG, LMPG) and 1-palmitoyl-2-hydroxy-*sn*-glycero-3-phospho-(1′-*rac*-glycerol) (16∶0 Lyso PG, LPPG) were purchased from Avanti Polar Lipids. Deuterium oxide (D_2_O) was purchased from Cambridge Isotope Labs and all other chemicals were obtained from Fisher Scientific, unless otherwise noted.

Several concentrations up to 200 mM were prepared for each detergent in a final buffer consisting of 20 mM phosphate buffer, pH 6.2, 150 mM NaCl, and 10% v/v D_2_O (necessary for the NMR deuterium lock). Detergent monomers do not contribute significantly to the observed scattering [22], and all scattering profiles presented are at concentrations well above the cmc (Table 1). Detergent concentrations were verified using 1D ^1^H-NMR and standards of known concentration.

Three molecular weight standards were used for the X-ray scattering experiments: hen egg white lysozyme in 40 mM acetate buffer, pH 3.8 with 150 mM NaCl, horse heart cytochrome c (Sigma) in 100 mM acetate buffer, pH 4.6 with 0.5 M guanidinium hydrochloride, and bovine serum albumin (Sigma) in 20 mM HEPES buffer, pH 7.8 with 50 mM NaCl. Five concentrations were measured for each protein standard (up to 10.6 mg/mL lysozyme, 4.2 mg/mL cytochrome c, and 8.6 mg/mL albumin) to determine any concentration dependent effects on the scattering. The scattering profiles that did not demonstrate concentration dependent changes were used to calculate *κ*, the proportionality constant in Equation 6 (see Methods S1) that is used to determine micelle aggregation numbers.

### SAXS Data Collection

SAXS data were measured at the XOR/BESSRC undulator beam line 12-ID-B of the Advanced Photon Source (Argonne, IL), with a sample-to-detector distance of 2 m and a Pilatus 2 M detector. The data were collected at 25°C using a custom-made sample holder [23] and an X-ray energy of 12 keV (corresponding to a wavelength of *λ* = 1 Å). The useable range of momentum transfer *q* was 0.02<*q*<0.3 Å^−1^ (*q* = 4*π* sin(*θ*)/*λ*, where 2*θ* is the scattering angle and *λ* is the x-ray wavelength). Additional descriptions of the beamline setup and measurement are previously published [23]–[25].

For the protein molecular weight standard samples, five exposures of 0.1 s were collected, image corrected, and circularly averaged. For the detergent samples, at least five exposures of 0.5 s each were collected. The absence of radiation damage was confirmed by comparing subsequent exposures of the same sample with no significant changes in the SAXS profile detected (data not shown). The five resulting profiles for each condition were averaged to improve signal quality. Matched buffer profiles were collected using identical procedures and subtracted from the sample scattering for background correction.

**Figure 1:**
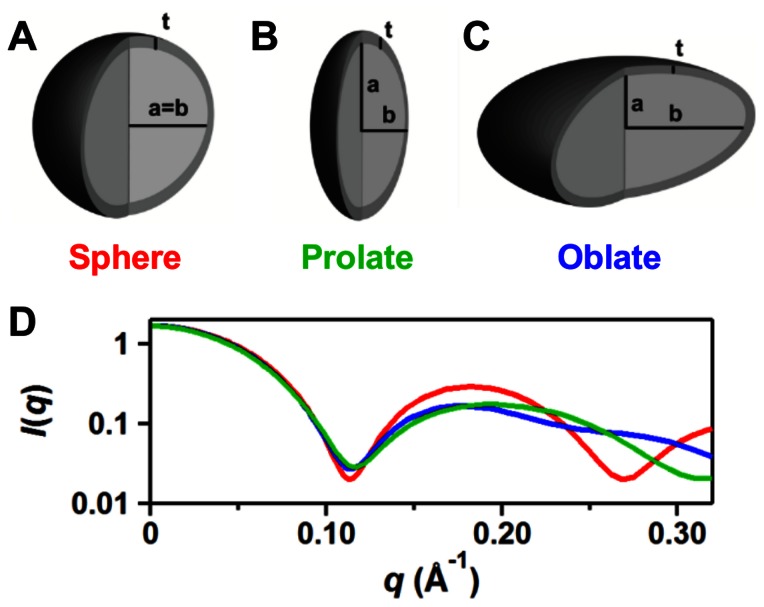
Two-component core-shell models used to represent the hydrophobic micelle core and hydrophilic head group shell. Schematic of the core-shell models having core axial dimensions of *a* and *b* with a uniform shell thickness of *t* for a (A) sphere (a = b), (B) prolate ellipsoid (a>b), and (C) oblate ellipsoid (a<b). The alkyl chain core is typically less electron dense (0.27–0.29 e/Å^3^) than the solvent (0.33–0.34 e/Å^3^) and the detergent head group shell (0.49–0.52 e/Å^3^). These differences in scattering power, along with size and shape of the micelle determine the form factors of the SAXS profile (see Methods S1 for additional descriptions of SAXS theory and core-shell ellipsoid micelle models). Panel (D) shows a comparison of the expected form factors resulting from each of the three different model geometries: prolate ellipsoid in green (a/b = 2); oblate ellipsoid in blue (b/a = 2); and sphere in red (a/b = 1). Core and shell volumes, and electron densities (*ρ*_1_ = 0.28, *ρ*_2_ = 0.50, *ρ*_s_ = 0.337) were kept constant; sphere, *a* = *b* = 20.0 Å, *t* = 5.0 Å; prolate ellipsoid, *a* = 31.7 Å, *b* = 15.9 Å, *t* = 4.8 Å; oblate ellipsoid, *a* = 12.6 Å, *b* = 25.2 Å, *t* = 4.8 Å.

Analysis of the SAXS profiles for each sample followed the procedures outlined in Lipfert, et al. [22] for determination of micelle size and shape parameters (Methods S1). One noted exception was the use of a nonlinear, least-square fitting routine implemented in Igor Pro (WaveMetrics) as part of the NCNR analysis toolkit [26] to fit the two component (core-shell) models to the full scattering profiles (e.g. sphere, oblate, and prolate; Fig. 1). Although designed to model fits to small-angle neutron scattering (SANS) data, this procedure was readily adapted to SAXS data by replacing scattering length densities (effective atomic scattering powers in SANS) with electron densities for the micelle core and shell components. Agreement between model-independent and similar model-derived values was used for additional validation of the modeling approach.

## Results and Discussion

**Figure 2:**
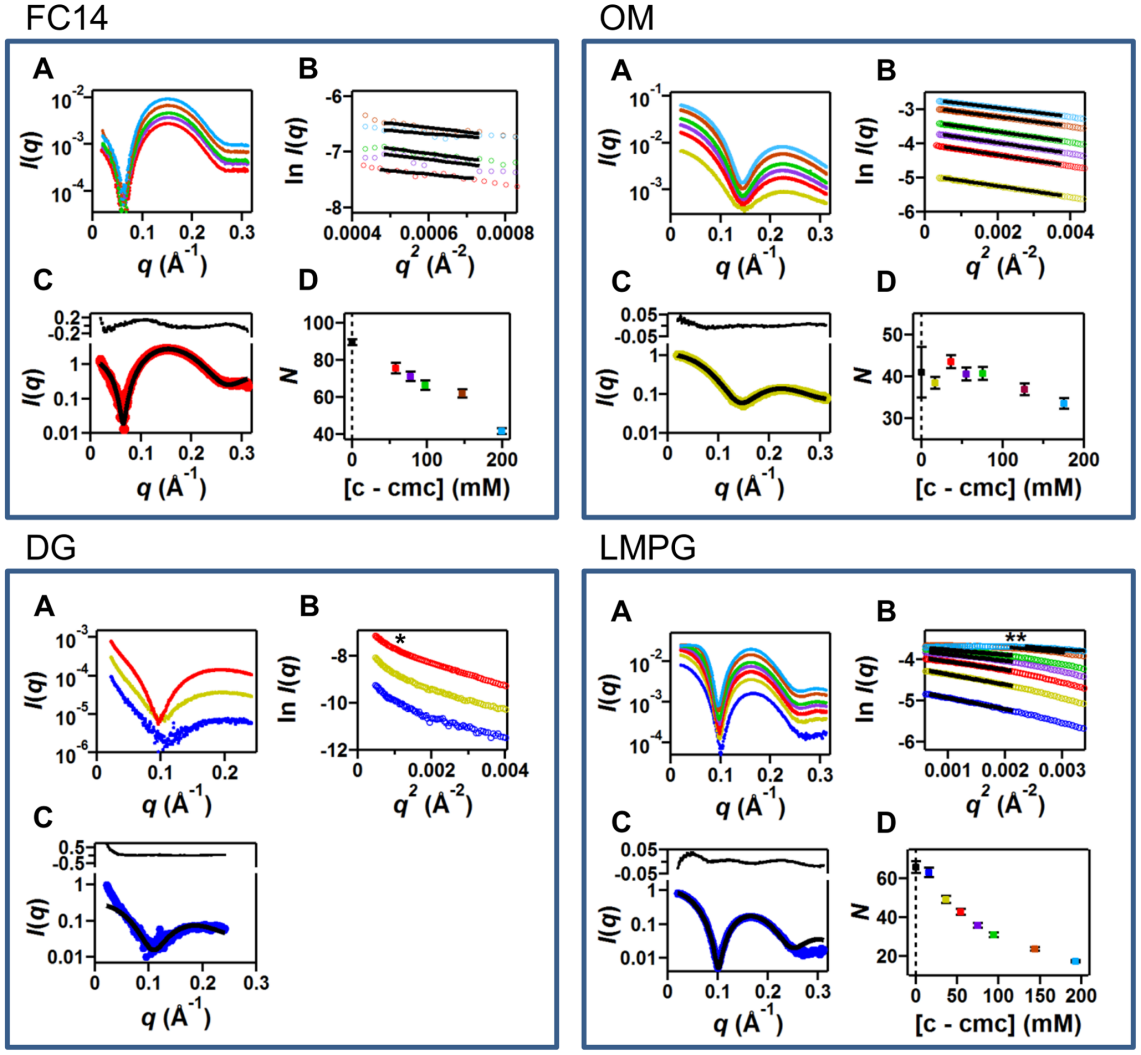
Scattering data, Guinier analysis, and core-shell ellipsoid fit for FC14, OM, DG, and LMPG. (A) SAXS profiles (*I*(*q*)) for each detergent at total detergent concentrations of FC14: 58 (red), 78 (purple), 97 (green), 147 (brown), and 199 (cyan) mM; OM: 36 (yellow), 56 (red), 75 (purple), 95 (green), 146 (brown), and 195 (cyan) mM; DG: 12.5 (blue), 25 (yellow), and 50 (red) mM; and LMPG: 16 (blue), 36 (yellow), 54 (red), 75 (purple), 94 (green), 144 (brown), and 193 (cyan) mM. (B) Guinier plot (ln(*I*) as a function of *q* ^2^) of the low angle data (same color scheme as part A) and Guinier fits (black lines). For DG, the Guinier region deviated from linearity at low *q* (denoted by *) as described in the results, and prevented accurate extrapolation to zero-scattering angle. Thus, aggregation numbers for DG could not be determined from the *I*(*0*) method, and the plot (panel D, see below) is not presented. An increase in scattering signal with increasing concentration is generally observed, except for LMPG at high concentration and low *q* (denoted with **) where interparticle effects described in the results become more apparent at higher concentrations. (C) Two-component ellipsoid fit (black solid line) and scattering intensity recorded at low detergent concentration (same color scheme as part A). The residuals of the fit are shown in the upper inset. Fit parameters are presented in Table 2. (D) Apparent aggregation numbers *N_expt_* (squares, same color scheme as in part A) obtained from the extrapolated forward scattering intensity and eq 7 described in Methods S1. The point at 0 mM (black) corresponds to the estimate obtained by linearly extrapolating the measured profiles for [*c*–cmc] ≤100 mM to zero micelle concentration (i.e. cmc). Errors are obtained from repeat fits using measurements from three molecular weight standards. Additional descriptions of micelle measurements and calculations can be found in Methods S1.

Eleven micelle-forming detergents, classified by head group structure, were chosen for this study: zwitterionic phosphocholines with 10-, 12-, and 14-carbon alkyl chains, nonionic glucosides with 8-, 9-, and 10-carbon alkyl chains, nonionic maltosides with 8-, 10-, and 12-carbon alkyl chains, and ionic lysophosphatidyl glycerols with 14- and 16-carbon alkyl chains (Table 1). At least one detergent studied from each of the four classes has demonstrated success in membrane protein structure determination. Micelle scattering data were previously published for some detergents from each class; however, to obtain meaningful trends in micelle size and shape with changes in chain length, additional detergents were studied. The concentration series of scattering profiles, Guinier plots, calculated aggregation numbers, and the best model fit to the lowest detergent concentration (to reduce contributions from interparticle interference) for the FC14, OM, DG, and LMPG are presented in Figure 2, while data showing agreement with the previously reported detergents [22] are presented in Figures S2, S3, S4, S5, S6, S7, and S8. The data, fits, and resulting shapes and sizes of each detergent are described in Results S1, where we also compare the results to the available literature. The physical parameters from model-independent (subscript *expt* is used) and dependent (subscript *model* is used) measurements for all the detergents are provided in Table 2 and Table S1.

**Table 2:** Geometrical parameters of detergent micelles.

detergent	shape	*ρ*_1_(e/Å^3^)	*ρ*_2_(e/Å^3^)	*a*(Å)	*b*(Å)	*t*(Å)	a/b	*Rg_expt_* (Å)	*Rg_model_* (Å)	*L_expt_* (Å)	*L_model_* (Å)	*N_expt_*	*N_model_*
FC10 (59 mM)	prolate	0.273	0.490	20.4–20.9	13.3–13.6	2.7–3.0	1.52–1.55	25.9±0.2	24.2±0.5	27.6–28.2	29.3–30.2	39–45	50–56
FC12 (77 mM)	prolate	0.277	0.490	24.3–24.8	16.1–16.4	2.7–3.0	1.49–1.52	34.5±0.8	32.6±0.5	33.9–34.5	34.9–35.8	68–80	72–80
FC14 (97 mM)	prolate	0.280	0.490	29.6–30.1	18.8–19.1	2.7–3.0	1.57–1.60	50.2 a ±3.7	44.6±0.5	41.4–42.0	40.3–41.2	88–91	106–116
OG (50 mM)	oblate	0.268	0.540	10.6–11.4	20.6–21.4	2.9–3.5	0.51–0.55	29.6^b^ ±2.2	23.5±0.5	26.9–27.5	24.1–26.3	n.d.^b^	70–90
NG (50 mM)	oblate	0.271	0.540	12.1–12.9	20.7–21.5	2.9–3.5	0.58–0.62	34.2^b^ ±2.3	24.2±0.5	29.6–30.2	27.1–29.3	n.d.^b^	80–100
DG (50 mM)	oblate	0.273	0.540	13.5–14.3	22.6–23.4	2.9–3.5	0.60–0.64	n.d.^b^	27.5±0.5	32.1–32.7	29.9–32.1	n.d.^b^	100–120
OM (56 mM)	oblate	0.268	0.520	11.0–11.4	18.4–18.8	5.4–5.8	0.59–0.61	22.0±0.1	21.1±0.5	27.9–28.5	27.4–28.6	35–47	65–71
DM (80 mM)	oblate	0.273	0.520	13.4–13.8	22.7–23.1	5.4–5.8	0.59–0.61	26.2±0.1	25.6±0.5	33.4–34.0	32.2–33.4	86–103	98–104
DDM (94 mM)	oblate	0.277	0.520	15.7–16.1	27.9–28.3	5.4–5.8	0.56–0.58	31.8±0.1	30.7±0.5	39.4–40.0	36.8–38.0	135–149	145–155
LMPG (16 mM)	oblate	0.280	0.470	16.6–17.6	23.5–24.5	5.3–6.1	0.70–0.73	26.9±2.4	28.8±0.5	38.7–39.3	38.5–41.3	63–69	90–100
LPPG^21^ (25 mM)	oblate	0.281	0.470	19.0–20.0	28.4–29.4	5.3–6.1	0.67–0.70	35.7±2.5	34.5±0.5	45.2–45.8	43.3–46.1	160–170	140–150

a For FC14, an average *Rg_expt_* from the lower concentration data (≤150 mM) was used because deviation from linearity in the Guinier region was observed;

b The Guinier regions for the glucosides are mostly nonlinear in the range that q*Rg<1.3, however estimations were made from lower concentration data where possible. In addition, the rise in intensity as *q* 0 precluded the determination of forward scattering intensities and thus *N_expt_* could not be determined.

Parameters were obtained from optimal core-shell ellipsoid model fits to the experimental SAXS data at given total concentrations of detergent in solution.

In summary, the micelles were modeled using a two-component ellipsoid (Fig. 1) for which the ellipsoidal dimensions (*a*, *b*. and *t*), and the radius of gyration (*Rg_model_*) and aggregation number (*N_model_*) were determined (Table 2). In addition, model independent parameters (*Rg_expt_*, *N_expt_,*and *L_expt_*) were measured or calculated directly from the scattering profile or from Guinier analysis of the low *q* data (Table 2). For further description of the physical parameters and determination of each, see Methods S1. Micelle geometries (sphere, oblate/prolate ellipsoid) for each detergent were determined by comparing the fits for each shape to the experimental SAXS profile (Fig. S9) and considering any physical constraints imposed on the model. In addition, a cylindrical model was applied to the DG scattering profile (Fig. S10), but did not provide a more reasonable fit (see Results S1). The phosphocholine micelle models share a common 2.7–3.0 Å uniform shell thickness and a prolate ellipsoid geometry. The maltoside micelle models share a common 5.4–5.8 Å uniform shell thickness and an oblate ellipsoid geometry. The glucoside micelle models share a common 2.9–3.5 Å uniform shell thickness and an oblate ellipsoid geometry. Finally, the LPG micelle models share a common 5.3–6.1 Å uniform shell thickness and an oblate ellipsoid geometry. All detergent micelle models have a minor core axis approximating the length of a detergent monomer with almost fully extended alkyl chains. The four detergent types with multiple alkyl chain lengths provided a foundation for a systematic investigation of the trends in micelle size and shape with varied detergent properties.

### Comparison of the Two-component Ellipsoid Models and Model-free Derived Parameters

In order to validate the core-shell ellipsoid micelle model fits to the experimental data, comparisons were made between parameters that could be determined both from the geometric model and directly from the scattering profile (model-independent). The agreement between the model-independent radius of gyration, aggregation number, and dominant head group to head group length and that of the models strongly support the proposed ellipsoid models.

#### Radius of gyration (*Rg*)

**Figure 3:**
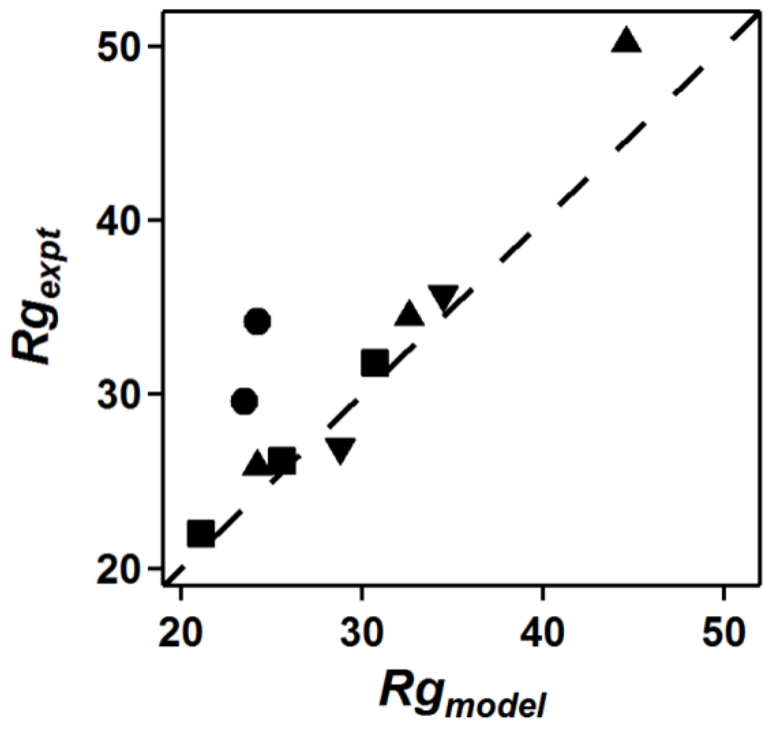
Radii of gyration obtained from Guinier fits and the ellipsoid models correlate. Radii of gyration (*Rg*’s) for varied alkyl chain lengths of phosphocholine (▴), glucoside (•), maltoside (▪), and lysophosphatidyl glycerol (▾) detergent head groups were determined from Guinier analysis of the low angle scattering data and calculated from the model geometry (Methods S1). A correlation plot illustrates the agreement between the *Rg* determined from the Guinier analysis of the scattering data (*Rg_expt_*) with the *Rg* calculated from the model geometry (*Rg_model_*) for each detergent. The dashed line represents a perfect correlation between the two approaches.

The radii of gyration calculated from the geometric model (*Rg_model_*) are near or within the reported error from the Guinier analysis of the experimental data (*Rg_expt_*) for all the detergents except the glucosides and FC14 (Table 2 and Fig. 3). However, evaluation of the values derived from the Guinier analysis must be taken with care as the scattering profile can be altered by attractive and/or repulsive effects between micelles (particularly at high concentrations), complicating accurate calculation of *I*(*0*) and *Rg*. In the case of FC14 and the glucosides, where there is some disagreement between the two *Rg* values, deviation from linearity in the low *q* region of the Guinier plots indicates micelle interactions that lead to an over-estimation of the size of the micelle.

#### Aggregation number (*N*)

**Figure 4:**
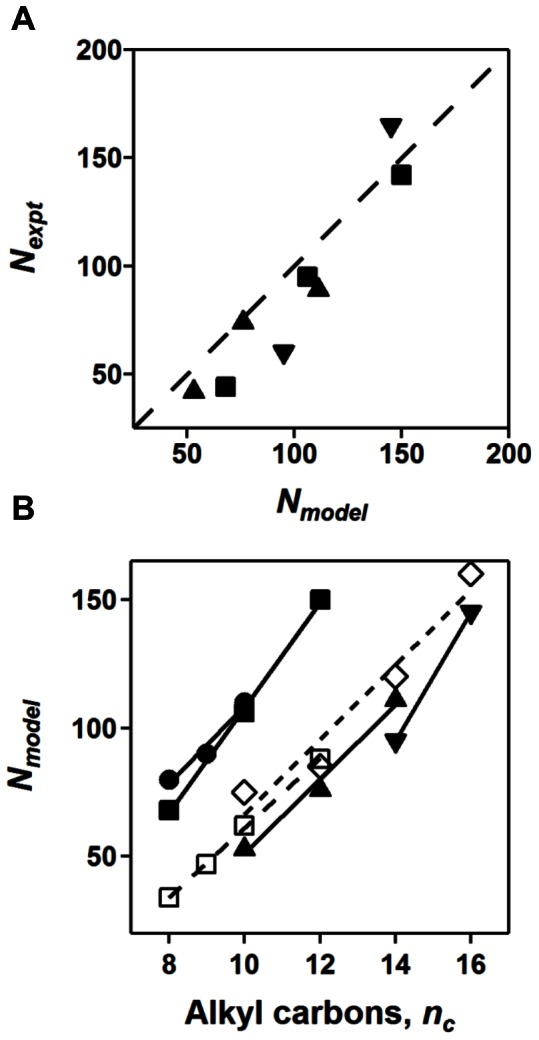
Dependence of aggregation number on alkyl chain length. (A) The relationship between aggregation numbers determined from hydrophobic core volume (*N_model_*) and Guinier analysis (*N_expt_*) is shown using same shapes for phosphocholine (▴), maltoside (▪), and lysophosphatidyl glycerol (▾) head groups with a dashed line illustrating *N_model_* = *N_expt_*. (B) Aggregation numbers for each alkyl chain series using the same symbols as in (A), as well as glucoside (•), (N-alkylamino)-1-deoxylactitol [30] (□), and sucrose ester [31] (◊) head groups, are plotted against the number of carbons comprising the alkyl chain. Solid lines (and dashed line for sucrose esters and lactitols) are linear fits to each data series, calculated from the hydrophobic core volume (*N_model_*). Equations and quality of fit are as follows: (▴), N = 14.5 *n_c_* –94, R^2^ = 0.986; (•), N = 15.0 *n_c_* –42, R^2^ = 0.964; (▪), N = 20.5 *n_c_* –97, R^2^ = 0.998; (□), N = 13.5 *n_c_* –75, R^2^ = 0.999; (◊), N = 14.5 *n_c_* –79, R^2^ = 0.944. Lysophosphatidyl glycerols fits are not reported since there are only two data points in the series.

Aggregation numbers calculated from the hydrophobic core volume of the micelle model (*N_model_*) were consistently larger (by about 10 monomers per micelle) than the aggregation numbers determined from the extrapolated forward scattering intensity (*N_expt_*) (Table 2 and Fig. 4A). This discrepancy between the aggregation numbers may be attributed to the additional separation between monomers, or monomer dynamics, in the micelle that is not accounted for in the direct volume calculation; that is, closest packing is assumed in the calculation. Although the geometric model parameters showed negligible concentration dependence (data not shown), a concentration dependence was observed in *N_expt_* (Fig. 2D) and *Rg_expt_* (data not shown), which was more pronounced in the charged micelles. The aggregation number from the forward scattering represents the value extrapolated back to the cmc concentration (zero micelle concentration) to provide an estimate free of concentration-dependent effects. The agreement of the experimentally determined aggregation numbers with the values reported in the literature further support the validity of the models. However, it should be noted that while the aggregation numbers determined for OM are consistent with the linear trends observed for the dependence of aggregation number on alkyl chain length (discussed below), these values do not agree with the available data from Affymetrix, Inc. (Table 1). Aggregation numbers from the forward scattering intensity are not reported for the glucoside detergents in this study (due to unreliable extrapolations of scattered intensity to zero scattering angle), and uncertainties in the wide range of published values provide only a qualitative comparison.

#### Dominant head group to head group length (*L*)

**Figure 5:**
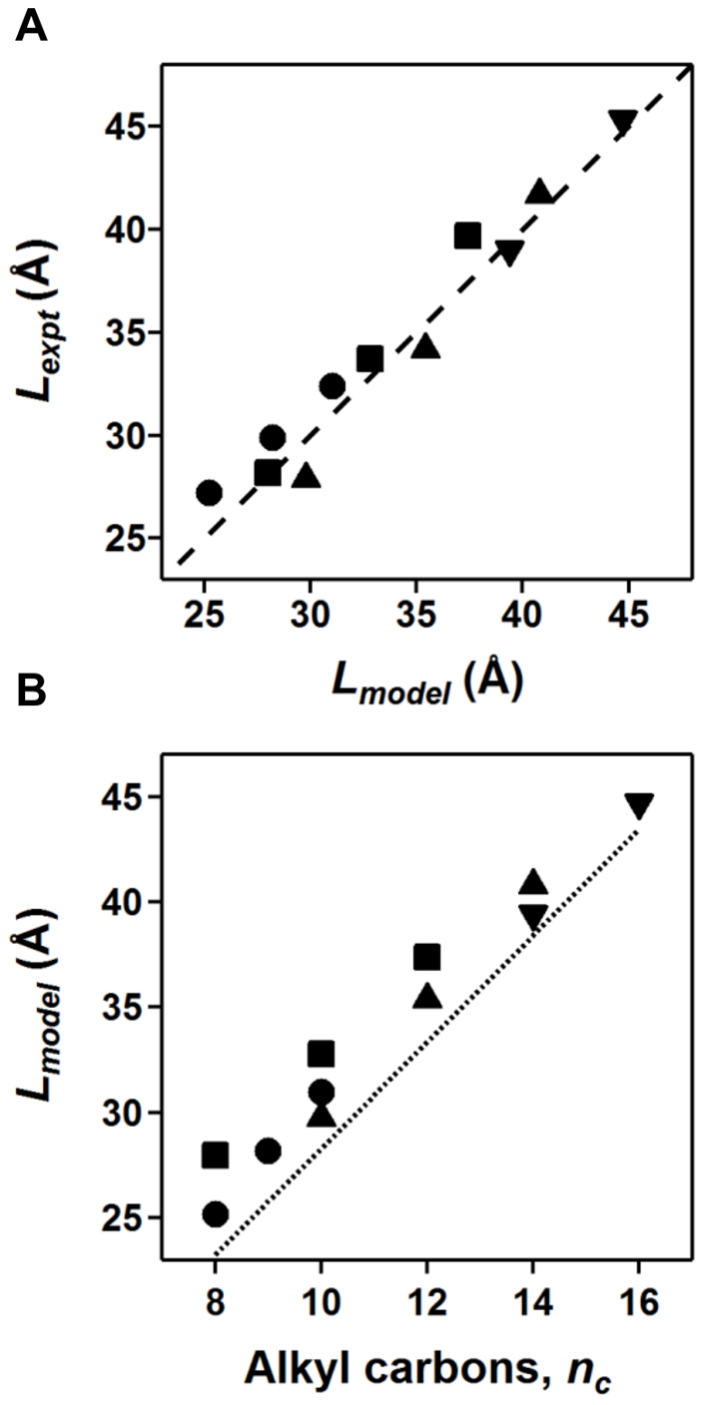
Dependence of dominant distance between head groups across the micelle on alkyl chain length. The dominant distances between head groups across the micelle are shown for phosphocholine (▴), glucoside (•), maltoside (▪), and lysophosphatidyl glycerol (▾) detergents. (A) The correlation is shown between the dominant distance determined from the position of the second peak in the experimental SAXS profiles (*L_expt_*) and the corresponding distance estimated from the model fit (*L_model_*). The dashed line represents a perfect correlation between the two approaches. (B) The distance determined from the best model fit for each detergent (*L_model_*) is plotted as a function of the number of carbons in the detergent’s alkyl chain (*n_c_*). The dotted line represents the distance of two alkyl chains having a fully extended hydrocarbon chain according to Tanford’s formula for alkyl chain length.

The dominant head group to head group length describes the most frequently occurring distance between head groups separated by a pair of alkyl chains across the micelle. The 2^nd^ peak in the scattering profile provides a model-free assessment of the most frequently observed length scale, correlating to this distance *L* [13], [22]. The length between opposing head groups from the model (*L_model_*) are mostly within error or slightly shorter (1–2 Å) than the corresponding distance determined from the position of the 2^nd^ peak in the SAXS profile (*L_expt_*), except for FC10 and FC12 which are 1–2 Å longer (Table 2 and Fig. 5A). A comparison between the two measured dominant head group to head group lengths was also useful in assessing the micelle model ellipsoid shape (oblate vs. prolate). Some fits to the scattering profiles did not agree with the structure of the detergent monomer and the observed *L_expt_*; thus, they were not considered accurate models. Additionally, the dominant head group to head group length (*L_expt_*) is measured at intermediate scattering angles and is not susceptible to the inter-particle interference observed at low *q*.

### Head Group – Head Group Distances Increase with Alkyl Chain Length

With the data from multiple detergents systematically varying in chain length, the dependence of micelle physical parameters on chain length and head group could be investigated. The dominant head group – head group distances as determined from the position of the second maximum in the scattering profile (*L_expt_*) are plotted as a function of alkyl chain length in Figure 5B. In each class of detergents studied, the dominant distance between head groups across the micelle increases by 2.5–3.0 Å with the addition of each carbon to the alkyl chain (note that the distance across the micelle contains two opposing alkyl chains). The short axial dimension of the fitted ellipsoid models similarly increases by 2.5–3.0 Å with each added alkyl carbon, while the thickness of the outer shell in the two-component is independent on alkyl chain length, as expected (Table 2). The short ellipsoid axis dimension increases by ∼2.5 Å for every two carbons added to the alkyl chain for the phosphocholines, ∼2.4 Å for every two carbons added to the alkyl chain for the maltosides, ∼1.5 Å for each carbon added to the alkyl chain for the glucosides, and ∼2.5 Å for the two carbon increase in the LPG tail length (Table 2). The average increase in alkyl chain length per added carbon unit for all micelles in this study is approximately 1.31±0.13 Å (length of short axis (*a* or *b*) divided by the number of carbons), which is in good agreement with the additional length expected by the addition of each carbon, ∼1.27 Å from Tanford’s formula [27] for the maximum extension of the alkyl chain (*l_c_ = *1.5+1.265 *n_c_*).

### Aggregation Numbers Increase with Carbon Chain Length

The aggregation numbers calculated from the model for each detergent are plotted as a function of alkyl chain length in Figure 4B, with the associated values listed in Table 2. The average increase from all detergent micelles in this study indicates that micelle aggregation numbers increase linearly by approximately 16±3 monomers per micelle with the addition of each carbon atom to the alkyl chain. This steady increase in aggregation number highlights the significant contributions to the increased hydrophobic interactions between adjacent monomers made by the addition to alkyl chain length.

Tanford predicted the maximal micelle aggregation numbers based on geometric packing of the alkyl chains in the hydrocarbon core for spherical and ellipsoidal micelles over a given range of ellipticities and chain lengths [28]. The detergent monomers in this case are assumed to have a maximum alkyl chain extension, which will result in overestimation of the aggregation number [29]. The best-fit model parameters for the phosphocholine data indicate a prolate ellipsoid geometry with *a/b* ∼1.5, which corresponds to a predicted increase of ∼14 monomers per micelle with each increased carbon in the alkyl chain, which is in agreement with the observed 12–15 monomers (Table 2 and Fig. 5B). The oblate maltosides (glucosides were excluded for reasons discussed above) have a relative a/b of ∼0.57 corresponding to a predicted increase of ∼28 monomers per micelle with each increased carbon in the alkyl chain [28] and in reasonable agreement with the observed 20–25 monomers. The oblate geometry with a relative a/b of ∼0.7 from the two LPG models corresponds to a predicted approximate increase of 20–25 monomers per micelle [28], which agrees well with the increase of 25 monomers per micelle determined in this study. In addition to aggregation number and core thickness, other measurements, such as radii of gyration, vary linearly with the number of carbons in the alkyl chain (1.55±0.85 Å per carbon added; Table 2).

Using the geometric packing approach of Tanford, the calculated values for the aggregation numbers (*e.g.* 60, 83, and 110 for *n_c_ = *10, 12, and 14, respectively, for phosphocholine; 47, 58, 70, and 97 for *n_c_* = 8, 9, 10, and 12, respectively, for maltosides; and 105 for *n_c_ = *14 and 134 for *n_c_ = *16 for LPG) are also in good agreement with values determined in this study (Table 2), given that the theoretical model likely presents an overestimation due to the assumed maximum extension of the alkyl chain. A comparison of the alkyl chain length predicted from the maximum extended chain length and determined from the micelle model indicates that the alkyl chains of the model are 80–90% of the maximum extension, consistent with a flexible yet closely-packed hydrocarbon core [27].

### Aggregation Number Decreases with Steric Bulk or Electrostatic Repulsion

In addition to chain length, a major determinant of micelle aggregation number is the size and charge of the head group. The aggregation numbers of the nonionic series depend on the head group identity as can be observed by comparing the aggregation numbers for a single chain length (Fig. 4). Two previously reported data sets for (N-alkylamino)-1-deoxylactitols [30] and sucrose esters [31] are included in Figure 4B, to provide additional comparisons of head group effects. The general trend observed is that with an increase in steric bulk (nearest to the alkyl chain; Fig. S11) the aggregation number decreases. The charged LPGs have even smaller aggregation numbers compared to that extrapolated for the polar sugar head groups. Phosphocholine prolate micelles cannot be directly compared based on head group to the oblate micelles because for the same chain length and aspect ratio, prolate micelles have a smaller aggregation number (larger surface area per head group) than the oblate micelle regardless of the repulsion that is likely to exist between the head groups [28].

### Micelle Shape and Ellipticity Depend on Head Group Packing

Micelle shapes have been debated in the literature [28], [31]–[35]; however, the overwhelming evidence indicates that for single chain detergents the head group properties dictate the ellipticity (aspect ratio; a/b) of the micelle and that prolate and oblate micelles are observed [22], [28], [30], [31], [36]. Beyond this simplified treatment of the head group interactions, Iyer and Blankschtein [37] and Dupuy *et al.* [30] propose models for non-ionic surfactants that predict ellipticity and shape based on the packing and interactions between head groups (and solvent) that are independent of chain length. Their model also predicts that oblate micelles are preferred for small nonionic detergents, but that as the head group size increases or electrostatic repulsion occurs prolate micelles may be the preferred. The oblate ellipticity is predicted to approach spherical (aspect ratio of 1) as the head group increases in size or has repulsive electrostatic interactions (larger surface area). Indeed, this trend is observed in the ellipticities determined for the glucosides, maltosides, and LPGs (Table 2); the electrostatic repulsions between negatively charged head groups in LPG micelles gives rise to a more spherical micelle geometry compared to the uncharged glucosides and maltosides. The zwitterionic phosphocholines form prolate micelles indicating that the head group has significant steric and electrostatic repulsion and has a high surface area. This effect may be exaggerated due to counterion interactions at the surface with the prediction that ellipticity of the phosphocholine prolate micelle would be dependent on ionic strength. Also, as predicted by the Iyer and Blankschtein model, the micelle ellipticity does not change with an increase in chain length and aggregation number (Table 2).

### Conclusions

The detergents investigated form micellar aggregates, which resemble core-shell ellipsoids, with near maximally extended alkyl chains comprising the core, and a compact shell formed by the detergent head groups which separates the hydrophobic core from the surrounding aqueous environment. Elliptical geometries provided the best fits to the micelle scattering data with dimensions that were consistent with the physical properties of the detergent monomer. The size of the micelles increased linearly with an increase in alkyl chain length; approximately 16±3 monomers per micelle with the addition of each carbon atom. The increase in size was accommodated by an increase in the longer axis; however only to the length that maintained the ratio of the axes. These results provide a better understanding of the principles of detergent self-assembly, which will allow predictions of other micelle properties based on these principles. In addition, these results provide a foundation of physical properties important to the understanding of mixed detergent and PDC systems.

### Supporting Information

Supplemental figures and tables are provided detailing the chemical structures of detergents, in addition to the SAXS profiles, Guinier plots, and calculated geometric parameters for each detergent micelle. Supplemental texts (Methods S1 and Results S1) describing additional details of the SAXS methodology and theory followed by a discussion of the results pertaining to each class of detergent micelle are also included as supporting information.

## Supporting Information

Figure S1 Chemical structures of micelle-forming detergents characterized by SAXS. Structures of phosphocholines with 10, 12, and 14 alkyl carbons (FC10/FC12/FC14), glucosides with 8, 9, and 10 alkyl carbons (OG/NG/DG), maltosides with 8, 10, and 12 alkyl carbons (OM/DM/DDM), and lyso-phosphatidyl glycerols with 14 and 16 alkyl carbons (LMPG/LPPG).
(TIF)

Figure S2 Scattering data, Guinier analysis, and two-shell ellipsoid fit for FC10. (A) SAXS profiles (*I*(*q*)) of FC10 at total detergent concentrations of 36 (yellow), 59 (red), 76 (purple), 96 (green), 145 (brown), and 194 (cyan) mM. (B) Guinier plot (ln(*I*) as a function of *q* ^2^) of the very low angle data (same color code as part A) and Guinier fits (black lines). An increase in scattering signal with increasing concentration is observed. (C) Apparent aggregation numbers *N* obtained from the extrapolated forward scattering intensity and eq 7 (squares, same color code as in part A). The point at 0 mM (black) corresponds to the estimate obtained by linearly extrapolating the measured profiles for [*c*–cmc] ≤100 mM to zero micelle concentration (i.e. cmc). Errors are obtained from repeat fits using measurements from three molecular weight standards. (D) Two-component ellipsoid fit (black solid line) and scattering intensity recorded at a detergent concentration of 36 mM (yellow, as before). The residuals of the fit are shown in the upper inset. Fit parameters are presented in Table 2.
(TIF)

Figure S3 Scattering data, Guinier analysis, and two-shell ellipsoid fit for FC12. (A) SAXS profiles (*I*(*q*)) of FC12 at total detergent concentrations of 36 (yellow), 58 (red), 77 (purple), 95 (green), 145 (brown), and 194 (cyan) mM. (B) Guinier plot (ln(*I*) as a function of *q* ^2^) of the very low angle data (same color code as part A) and Guinier fits (black lines). An increase in scattering signal with increasing concentration is observed. (C) Apparent aggregation numbers *N* obtained from the extrapolated forward scattering intensity and eq 7 (squares, same color code as in part A). The point at 0 mM (black) corresponds to the estimate obtained by linearly extrapolating the measured profiles for [*c*–cmc] ≤100 mM to zero micelle concentration (i.e. cmc). Errors are obtained from repeat fits using measurements from three molecular weight standards. (D) Two-component ellipsoid fit (black solid line) and scattering intensity recorded at a detergent concentration of 36 mM (yellow, as before). The residuals of the fit are shown in the upper inset. Fit parameters are presented in Table 2.
(TIF)

Figure S4 Scattering data, Guinier analysis, and two-shell ellipsoid fit for DM. (A) SAXS profiles (*I*(*q*)) of DM at total detergent concentrations of 15 (blue), 36 (yellow), 58 (red), 80 (purple), 99 (green), 152 (brown), and 204 (cyan) mM. (B) Guinier plot (ln(*I*) as a function of *q* ^2^) of the very low angle data (same color code as part A) and Guinier fits (black lines). An increase in scattering signal with increasing concentration is observed. (C) Apparent aggregation numbers *N* obtained from the extrapolated forward scattering intensity and eq 7 (squares, same color code as in part A). The point at 0 mM (black) corresponds to the estimate obtained by linearly extrapolating the measured profiles for [*c*–cmc] ≤100 mM to zero micelle concentration (i.e. cmc). Errors are obtained from repeat fits using measurements from three molecular weight standards. (D) Two-component ellipsoid fit (black solid line) and scattering intensity recorded at a detergent concentration of 15 mM (blue, as before). The residuals of the fit are shown in the upper inset. Fit parameters are presented in Table 2.
(TIF)

Figure S5 Scattering data, Guinier analysis, and two-shell ellipsoid fit for DDM. (A) SAXS profiles (*I*(*q*)) of DDM at total detergent concentrations of 17 (blue), 32 (yellow), 57 (red), 75 (purple), 94 (green), 145 (brown), and 194 (cyan) mM. (B) Guinier plot (ln(*I*) as a function of *q* ^2^) of the very low angle data (same color code as part A) and Guinier fits (black lines). An increase in scattering signal with increasing concentration is observed. (C) Apparent aggregation numbers *N* obtained from the extrapolated forward scattering intensity and eq 7 (squares, same color code as in part A). The point at 0 mM (black) corresponds to the estimate obtained by linearly extrapolating the measured profiles for [*c*–cmc] ≤100 mM to zero micelle concentration (i.e. cmc). Errors are obtained from repeat fits using measurements from three molecular weight standards. (D) Two-component ellipsoid fit (black solid line) and scattering intensity recorded at a detergent concentration of 17 mM (blue, as before). The residuals of the fit are shown in the upper inset. Fit parameters are presented in Table 2.
(TIF)

Figure S6 Scattering data, Guinier analysis, and two-shell ellipsoid fit for OG. (A) SAXS profiles (*I*(*q*)) of OG at total detergent concentrations of 50 (red), 75 (purple), 100 (green), and 150 (brown) mM. (B) Guinier plot (ln(*I*) as a function of *q* ^2^) of the very low angle data (same color code as part A). Aggregation numbers were unable to be determined from the *I*(*0*) method, and the plot is not presented. (C) Two-component ellipsoid fit (black solid line) and scattering intensity recorded at detergent concentration of 50 mM (red, as before). The residuals of the fit are shown in the upper inset. Fit parameters are presented in Table 2.
(TIF)

Figure S7 Scattering data, Guinier analysis, and two-shell ellipsoid fit for NG. (A) SAXS profiles (*I*(*q*)) of NG at total detergent concentrations of 25 (blue), 50 (red), 100 (green), and 200 (cyan) mM. (B) Guinier plot (ln(*I*) as a function of *q* ^2^) of the very low angle data (same color code as part A). Aggregation numbers were unable to be determined from the *I*(*0*) method, and the plot is not presented. (C) Two-component ellipsoid fit (black solid line) and scattering intensity recorded at detergent concentration of 25 mM (blue, as before). The residuals of the fit are shown in the upper inset. Fit parameters are presented in Table 2.
(TIF)

Figure S8 Scattering data, Guinier analysis, and two-shell ellipsoid fit for LPPG. (A) SAXS profiles (*I*(*q*)) of LPPG at total detergent concentrations of 5 (blue), 10 (yellow), 25 (red), 50 (purple), 100 (green), 150 (brown), and 200 (cyan) mM. (B) Guinier plot (ln(*I*) as a function of *q* ^2^) of the very low angle data (same color code as part A) and Guinier fits (black lines). An increase in scattering signal with increasing concentration is observed. Note that the Guinier region at high concentrations is highly distorted by interparticle repulsions between charged micelles. As this data was not collected with the same molecular weight standards, aggregation numbers determined from the forward scattering are not shown. (C) Two-component ellipsoid fit (black solid line) and scattering intensity recorded at detergent concentration of 5 mM (blue, as before). The residuals of the fit are shown in the upper inset. Fit parameters are presented in Table 2.
(TIF)

Figure S9 Best core-shell model fits using different geometries (oblate/prolate ellipsoid and sphere) to micelle scattering profiles for multiple alkyl chain lengths of each detergent class. (A) SAXS profiles (*I*(*q*)) of phosphocholine detergents (FC10: blue, FC12: green, and FC14: red) at total detergent monomer concentrations of 59, 77, and 97 mM (corresponding to ∼1 mM of micelle), respectively, with the best model fits for each geometry shown as black lines. Residuals are shown above for each alkyl chain length using the same color coding. Similar comparisons are shown for: (B) maltoside detergents (OM: blue, DM: green, and DDM: red) at total monomer concentrations of 56, 80, and 94 mM, respectively, (C) glucoside detergents (OG: blue, NG: green, and DG: red) at total monomer concentrations of 50 mM, and (D) lysophosphatidyl glycerol detergents (LMPG: blue and LPPG: red) at total monomer concentrations of 16 and 25 mM, respectively.
(TIF)

Figure S10 Comparison of sphere (red), oblate ellipse (blue), and cylinder (cyan) geometrical model fits to OG scattering data. The sphere model contains a core radius of 17.1 Å with a shell thickness of 3.6 Å. The ellipse model is oblate and contains a core with semi-axes of 21.4 Å and 10.5 Å with a shell thickness of 3.1 Å. The cylinder model has a core radius of 12.3 Å with a shell thickness of 2.9 Å, and total length of 92.9 Å. Core, shell, and solvent contrasts were consistent between the models.
(TIF)

Figure S11 Head groups of each of the nonionic detergents compared in Figure 4. The atom attached to the alkyl chain is indicated by an arrow. The head groups are in order of increasing steric bulk from left to right (glucose<maltose<lactitol ≈ sucrose ester).
(TIF)

Table S1 Values of geometric model fits to the scattering profiles for each detergent studied classified by model shape (oblate/prolate ellipsoid and sphere).
(DOCX)

Methods S1 Supporting information for methods of determining micelle size and shape from small angle X-ray scattering data.
(DOCX)

Results S1 Supporting information and results of micelle physical properties with comparison to relevant studies.
(DOCX)
