# Supplementary figures and images for: Dependence of Micelle Size and Shape on Detergent Alkyl Chain Length and Head Group
Source: PLoS One. 2013 May 8;8(5):e62488. doi: 10.1371/journal.pone.0062488 (PMC3648574; doi:10.1371/journal.pone.0062488)

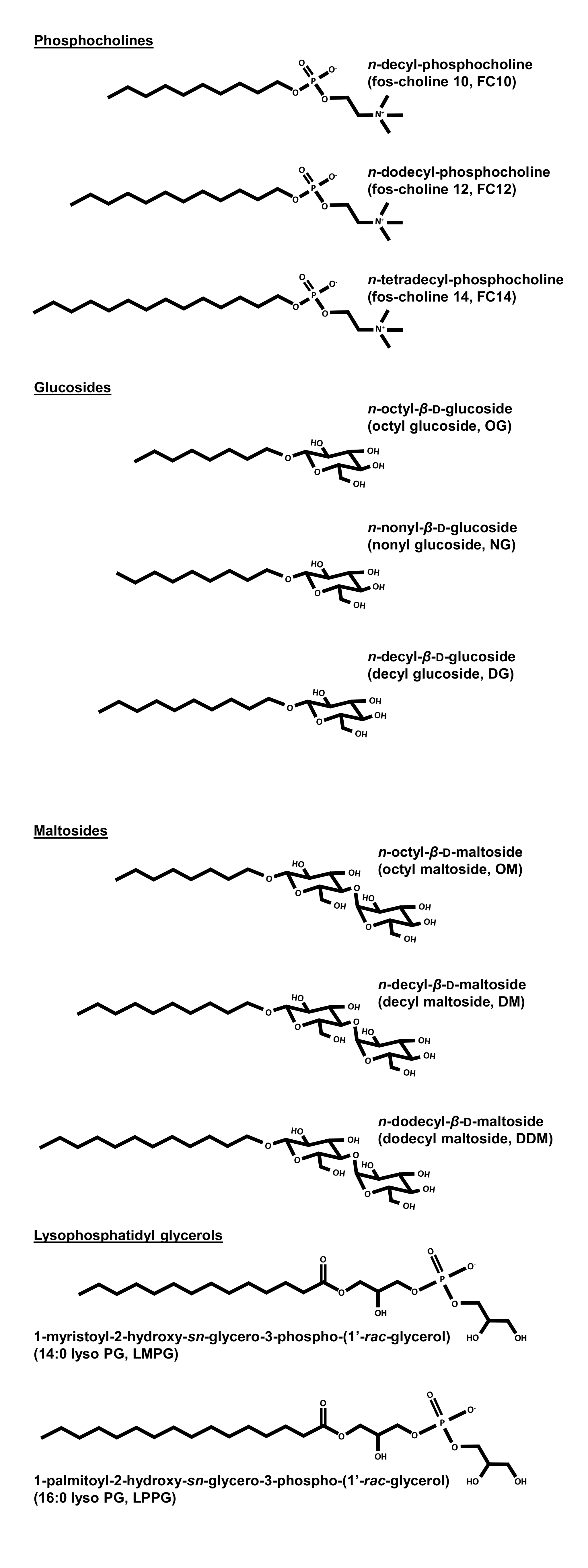

Supplement: Figure S1 — Chemical structures of micelle-forming detergents characterized by SAXS. Structures of phosphocholines with 10, 12, and 14 alkyl carbons (FC10/FC12/FC14), glucosides with 8, 9, and 10 alkyl carbons (OG/NG/DG), maltosides with 8, 10, and 12 alkyl carbons (OM/DM/DDM), and lyso-phosphatidyl glycerols with 14 and 16 alkyl carbons (LMPG/LPPG). (TIF) [file pone.0062488.s001.tif]

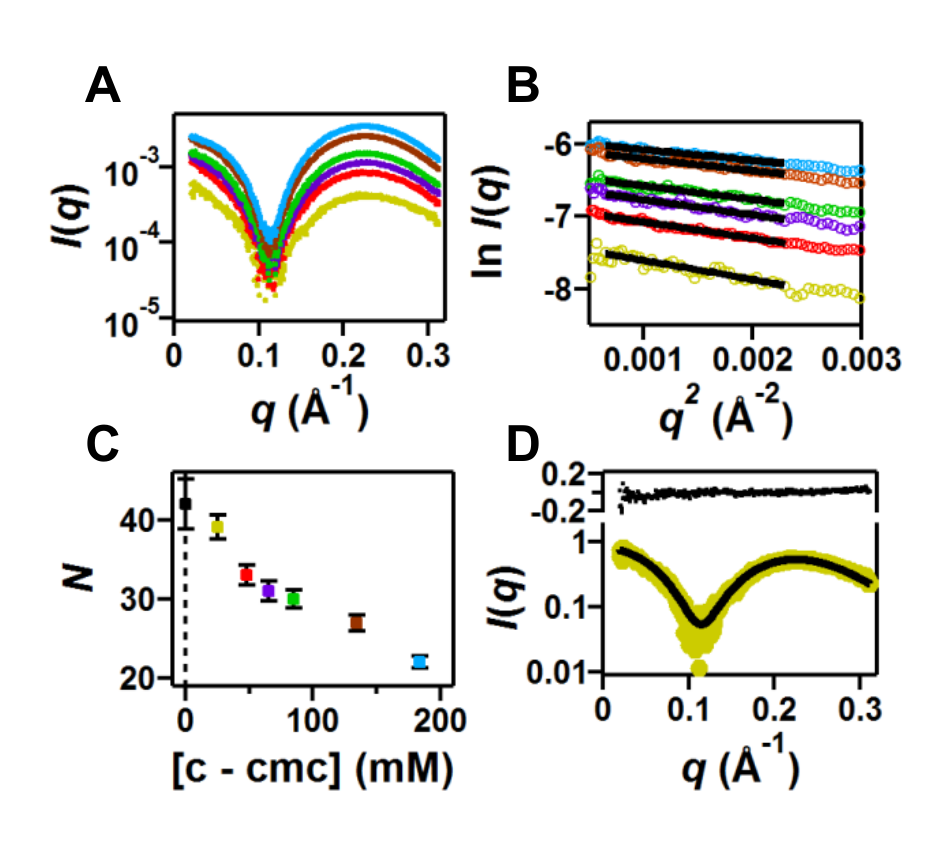

Supplement: Figure S2 — Scattering data, Guinier analysis, and two-shell ellipsoid fit for FC10. (A) SAXS profiles (I(q)) of FC10 at total detergent concentrations of 36 (yellow), 59 (red), 76 (purple), 96 (green), 145 (brown), and 194 (cyan) mM. (B) Guinier plot (ln(I) as a function of q 2) of the very low angle data (same color code as part A) and Guinier fits (black lines). An increase in scattering signal with increasing concentration is observed. (C) Apparent aggregation numbers N obtained from the extrapolated forward scattering intensity and eq 7 (squares, same color code as in part A). The point at 0 mM (black) corresponds to the estimate obtained by linearly extrapolating the measured profiles for [c–cmc] ≤100 mM to zero micelle concentration (i.e. cmc). Errors are obtained from repeat fits using measurements from three molecular weight standards. (D) Two-component ellipsoid fit (black solid line) and scattering intensity recorded at a detergent concentration of 36 mM (yellow, as before). The residuals of the fit are shown in the upper inset. Fit parameters are presented in Table 2. (TIF) [file pone.0062488.s002.tif]

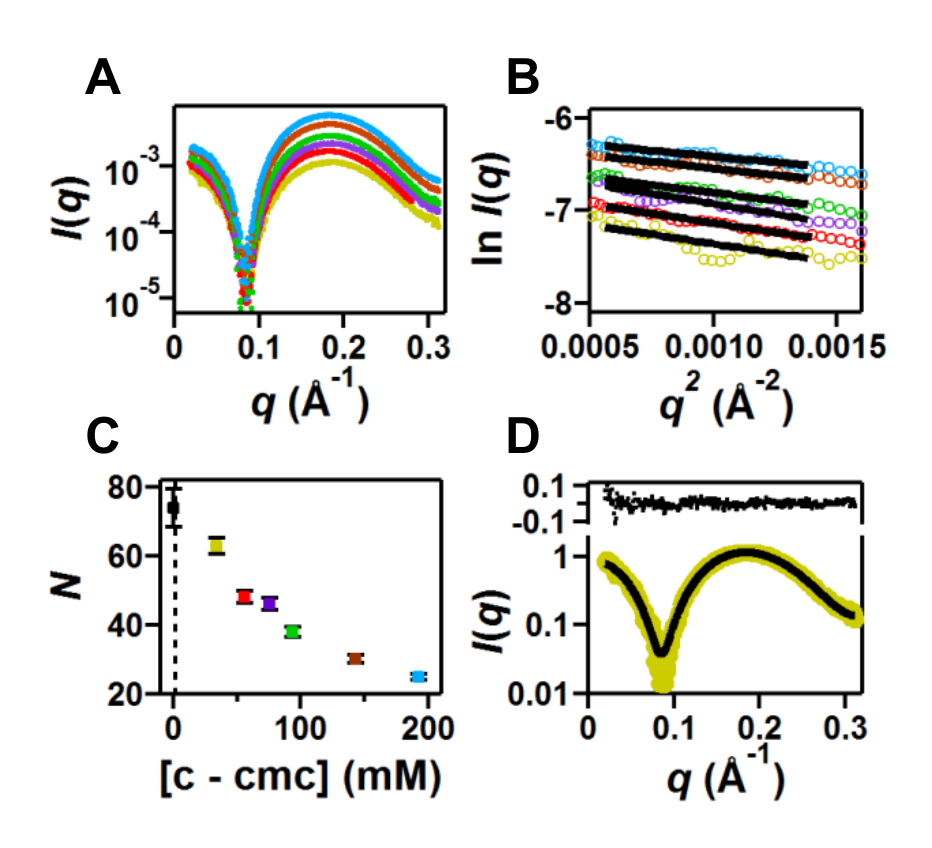

Supplement: Figure S3 — Scattering data, Guinier analysis, and two-shell ellipsoid fit for FC12. (A) SAXS profiles (I(q)) of FC12 at total detergent concentrations of 36 (yellow), 58 (red), 77 (purple), 95 (green), 145 (brown), and 194 (cyan) mM. (B) Guinier plot (ln(I) as a function of q 2) of the very low angle data (same color code as part A) and Guinier fits (black lines). An increase in scattering signal with increasing concentration is observed. (C) Apparent aggregation numbers N obtained from the extrapolated forward scattering intensity and eq 7 (squares, same color code as in part A). The point at 0 mM (black) corresponds to the estimate obtained by linearly extrapolating the measured profiles for [c–cmc] ≤100 mM to zero micelle concentration (i.e. cmc). Errors are obtained from repeat fits using measurements from three molecular weight standards. (D) Two-component ellipsoid fit (black solid line) and scattering intensity recorded at a detergent concentration of 36 mM (yellow, as before). The residuals of the fit are shown in the upper inset. Fit parameters are presented in Table 2. (TIF) [file pone.0062488.s003.tif]

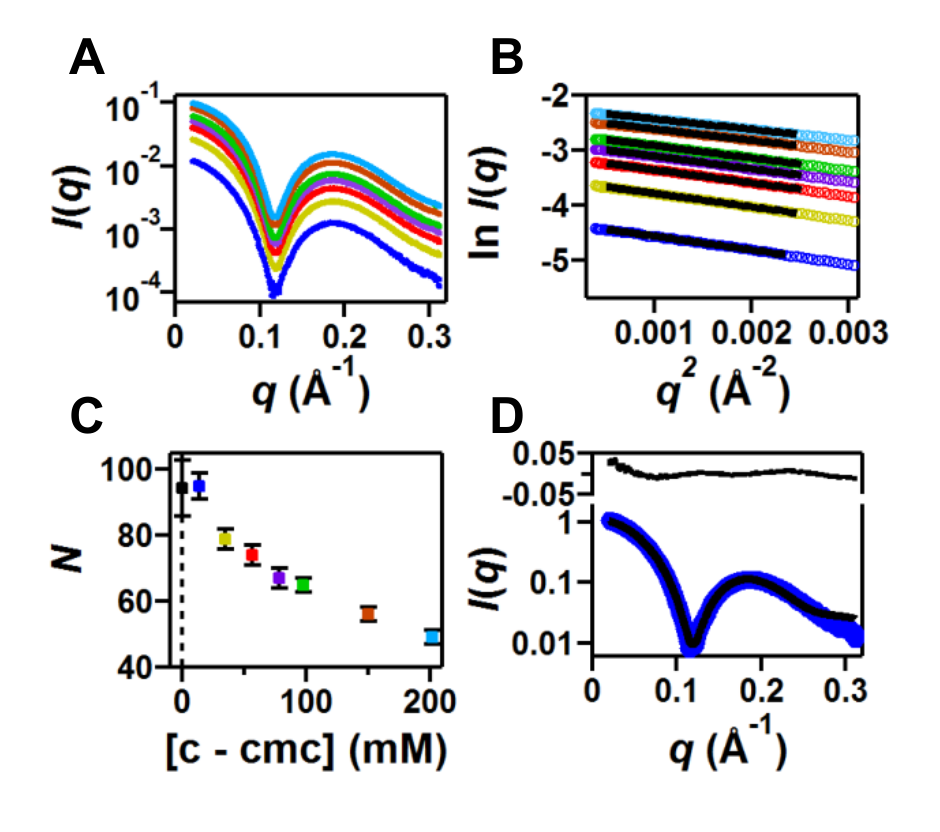

Supplement: Figure S4 — Scattering data, Guinier analysis, and two-shell ellipsoid fit for DM. (A) SAXS profiles (I(q)) of DM at total detergent concentrations of 15 (blue), 36 (yellow), 58 (red), 80 (purple), 99 (green), 152 (brown), and 204 (cyan) mM. (B) Guinier plot (ln(I) as a function of q 2) of the very low angle data (same color code as part A) and Guinier fits (black lines). An increase in scattering signal with increasing concentration is observed. (C) Apparent aggregation numbers N obtained from the extrapolated forward scattering intensity and eq 7 (squares, same color code as in part A). The point at 0 mM (black) corresponds to the estimate obtained by linearly extrapolating the measured profiles for [c–cmc] ≤100 mM to zero micelle concentration (i.e. cmc). Errors are obtained from repeat fits using measurements from three molecular weight standards. (D) Two-component ellipsoid fit (black solid line) and scattering intensity recorded at a detergent concentration of 15 mM (blue, as before). The residuals of the fit are shown in the upper inset. Fit parameters are presented in Table 2. (TIF) [file pone.0062488.s004.tif]

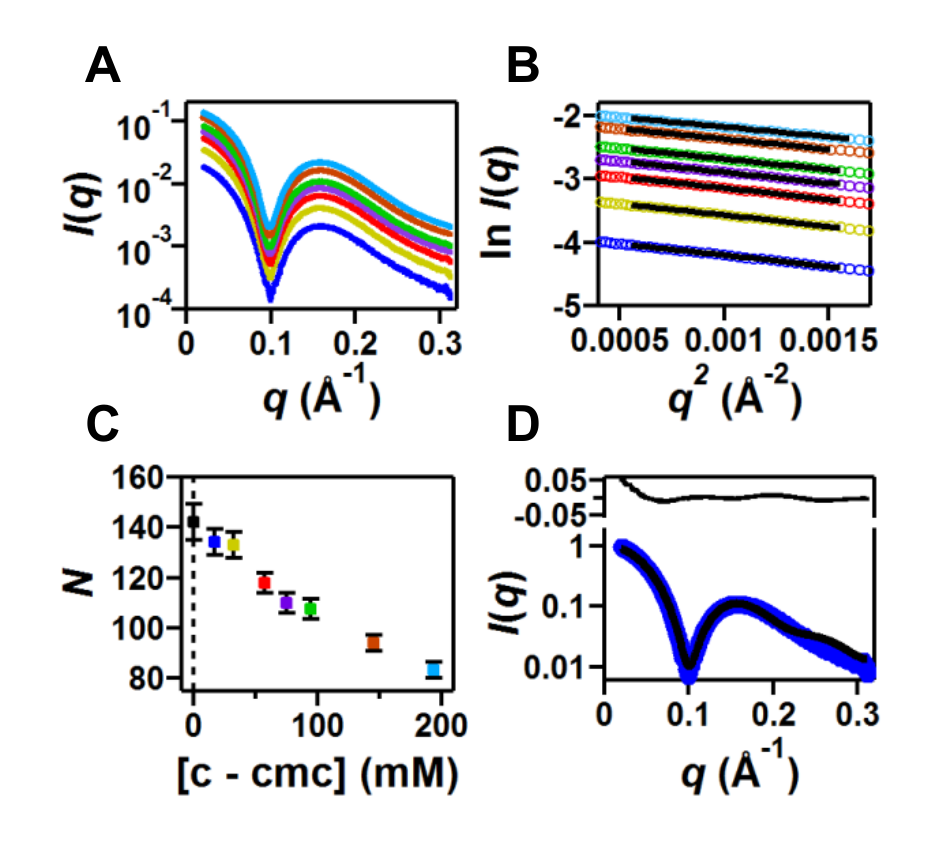

Supplement: Figure S5 — Scattering data, Guinier analysis, and two-shell ellipsoid fit for DDM. (A) SAXS profiles (I(q)) of DDM at total detergent concentrations of 17 (blue), 32 (yellow), 57 (red), 75 (purple), 94 (green), 145 (brown), and 194 (cyan) mM. (B) Guinier plot (ln(I) as a function of q 2) of the very low angle data (same color code as part A) and Guinier fits (black lines). An increase in scattering signal with increasing concentration is observed. (C) Apparent aggregation numbers N obtained from the extrapolated forward scattering intensity and eq 7 (squares, same color code as in part A). The point at 0 mM (black) corresponds to the estimate obtained by linearly extrapolating the measured profiles for [c–cmc] ≤100 mM to zero micelle concentration (i.e. cmc). Errors are obtained from repeat fits using measurements from three molecular weight standards. (D) Two-component ellipsoid fit (black solid line) and scattering intensity recorded at a detergent concentration of 17 mM (blue, as before). The residuals of the fit are shown in the upper inset. Fit parameters are presented in Table 2. (TIF) [file pone.0062488.s005.tif]

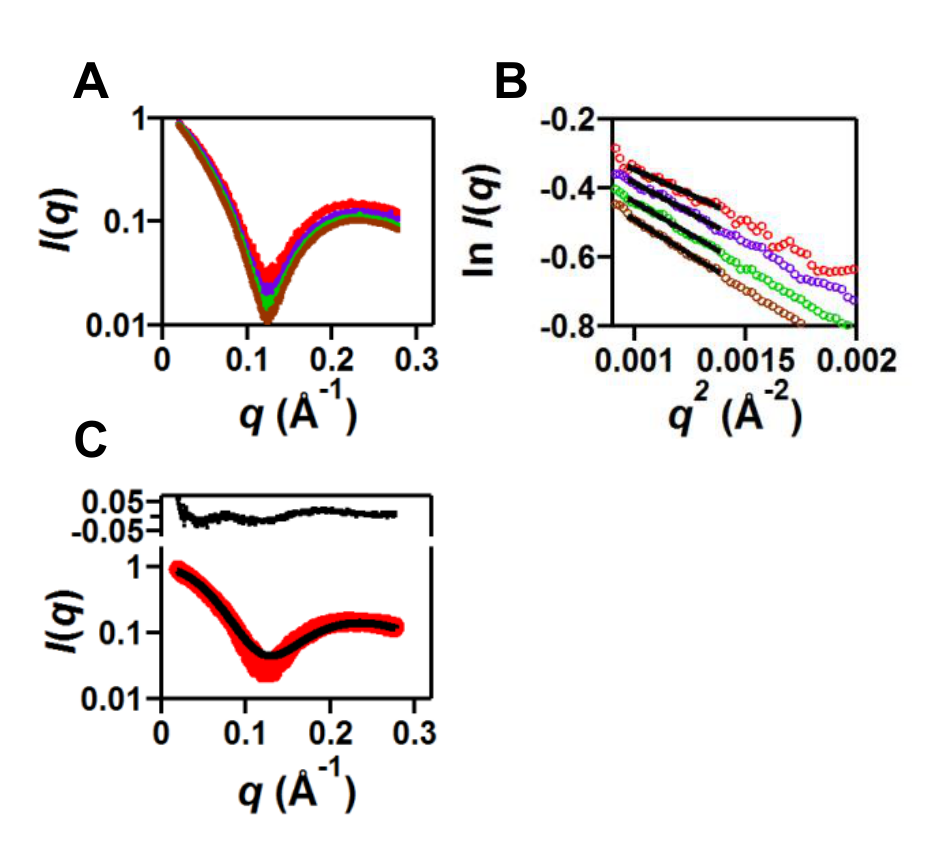

Supplement: Figure S6 — Scattering data, Guinier analysis, and two-shell ellipsoid fit for OG. (A) SAXS profiles (I(q)) of OG at total detergent concentrations of 50 (red), 75 (purple), 100 (green), and 150 (brown) mM. (B) Guinier plot (ln(I) as a function of q 2) of the very low angle data (same color code as part A). Aggregation numbers were unable to be determined from the I(0) method, and the plot is not presented. (C) Two-component ellipsoid fit (black solid line) and scattering intensity recorded at detergent concentration of 50 mM (red, as before). The residuals of the fit are shown in the upper inset. Fit parameters are presented in Table 2. (TIF) [file pone.0062488.s006.tif]

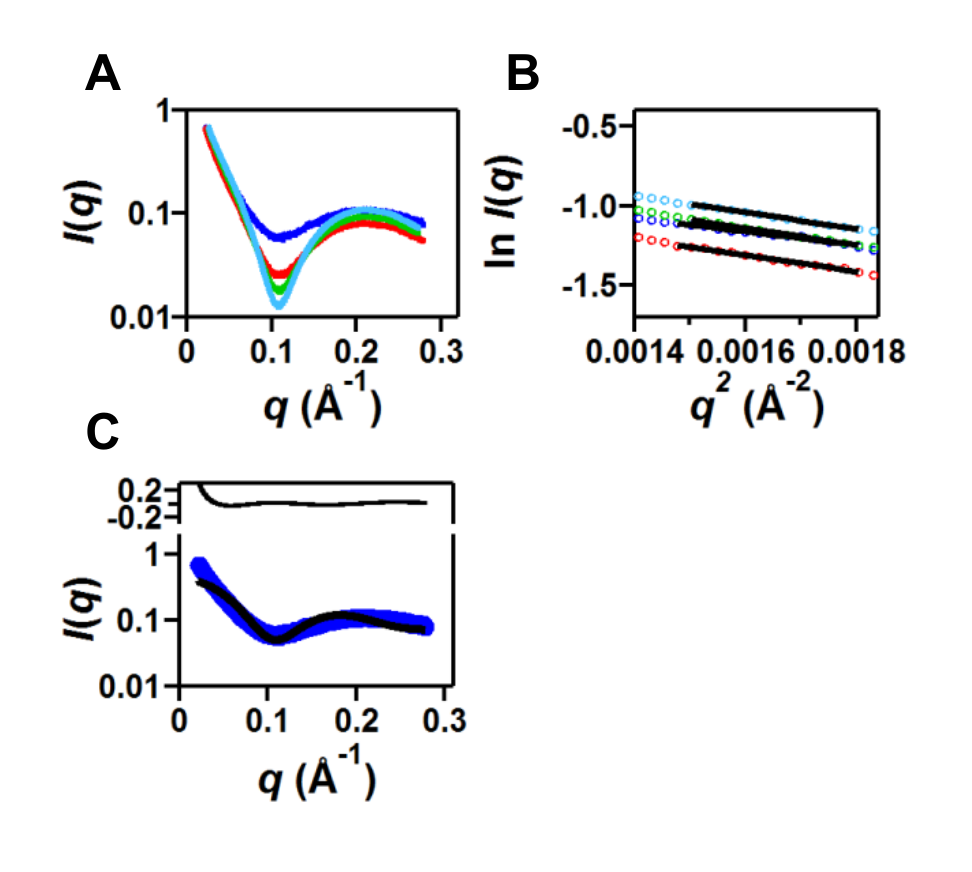

Supplement: Figure S7 — Scattering data, Guinier analysis, and two-shell ellipsoid fit for NG. (A) SAXS profiles (I(q)) of NG at total detergent concentrations of 25 (blue), 50 (red), 100 (green), and 200 (cyan) mM. (B) Guinier plot (ln(I) as a function of q 2) of the very low angle data (same color code as part A). Aggregation numbers were unable to be determined from the I(0) method, and the plot is not presented. (C) Two-component ellipsoid fit (black solid line) and scattering intensity recorded at detergent concentration of 25 mM (blue, as before). The residuals of the fit are shown in the upper inset. Fit parameters are presented in Table 2. (TIF) [file pone.0062488.s007.tif]

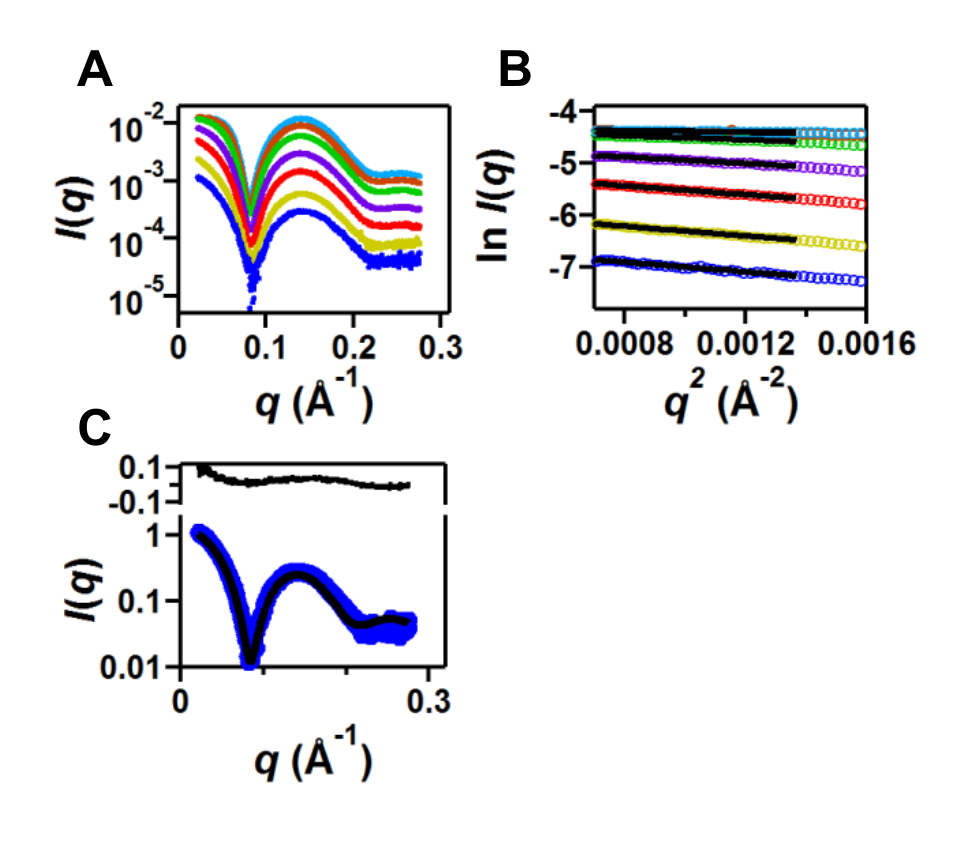

Supplement: Figure S8 — Scattering data, Guinier analysis, and two-shell ellipsoid fit for LPPG. (A) SAXS profiles (I(q)) of LPPG at total detergent concentrations of 5 (blue), 10 (yellow), 25 (red), 50 (purple), 100 (green), 150 (brown), and 200 (cyan) mM. (B) Guinier plot (ln(I) as a function of q 2) of the very low angle data (same color code as part A) and Guinier fits (black lines). An increase in scattering signal with increasing concentration is observed. Note that the Guinier region at high concentrations is highly distorted by interparticle repulsions between charged micelles. As this data was not collected with the same molecular weight standards, aggregation numbers determined from the forward scattering are not shown. (C) Two-component ellipsoid fit (black solid line) and scattering intensity recorded at detergent concentration of 5 mM (blue, as before). The residuals of the fit are shown in the upper inset. Fit parameters are presented in Table 2. (TIF) [file pone.0062488.s008.tif]

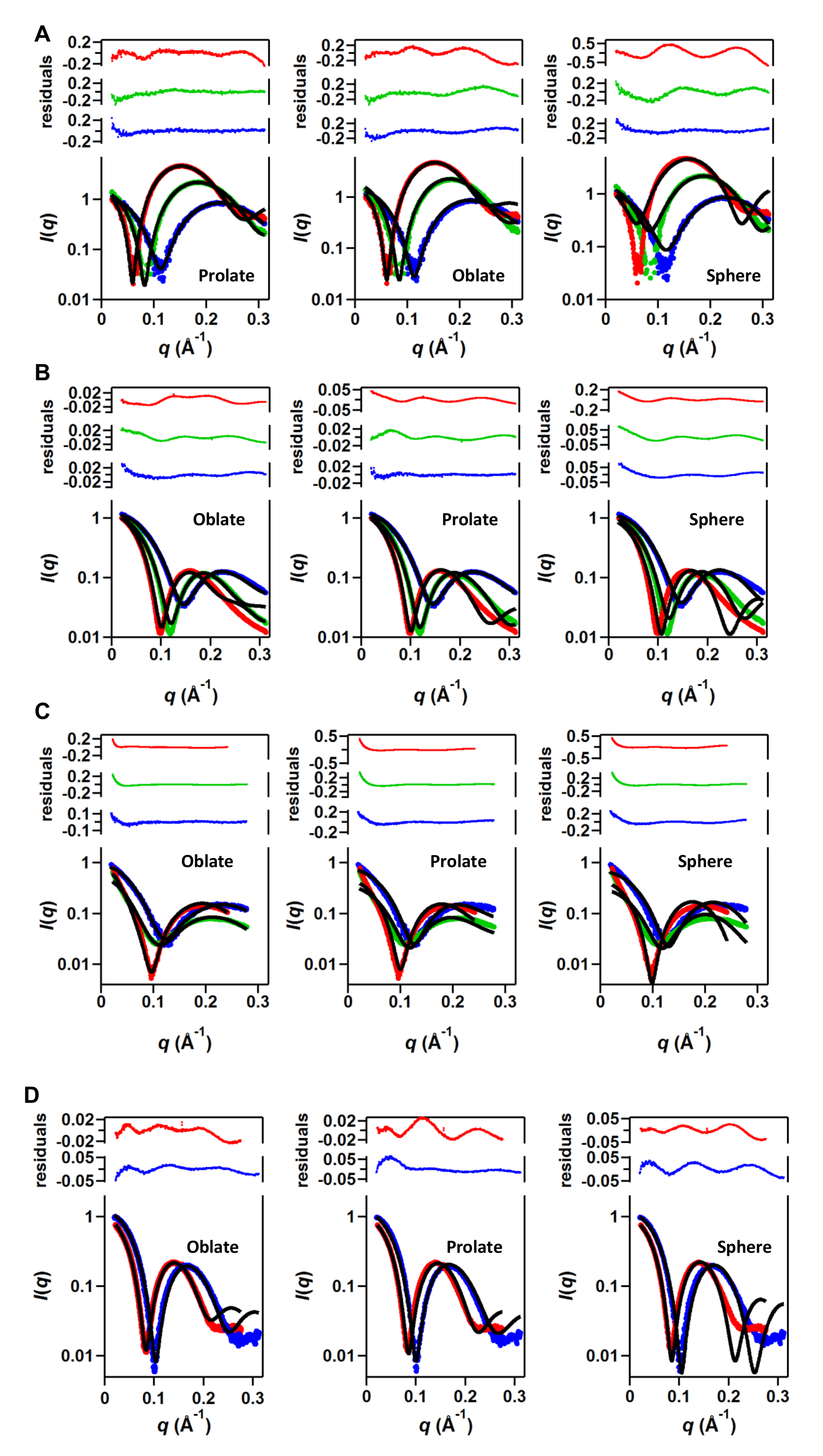

Supplement: Figure S9 — Best core-shell model fits using different geometries (oblate/prolate ellipsoid and sphere) to micelle scattering profiles for multiple alkyl chain lengths of each detergent class. (A) SAXS profiles (I(q)) of phosphocholine detergents (FC10: blue, FC12: green, and FC14: red) at total detergent monomer concentrations of 59, 77, and 97 mM (corresponding to ∼1 mM of micelle), respectively, with the best model fits for each geometry shown as black lines. Residuals are shown above for each alkyl chain length using the same color coding. Similar comparisons are shown for: (B) maltoside detergents (OM: blue, DM: green, and DDM: red) at total monomer concentrations of 56, 80, and 94 mM, respectively, (C) glucoside detergents (OG: blue, NG: green, and DG: red) at total monomer concentrations of 50 mM, and (D) lysophosphatidyl glycerol detergents (LMPG: blue and LPPG: red) at total monomer concentrations of 16 and 25 mM, respectively. (TIF) [file pone.0062488.s009.tif]

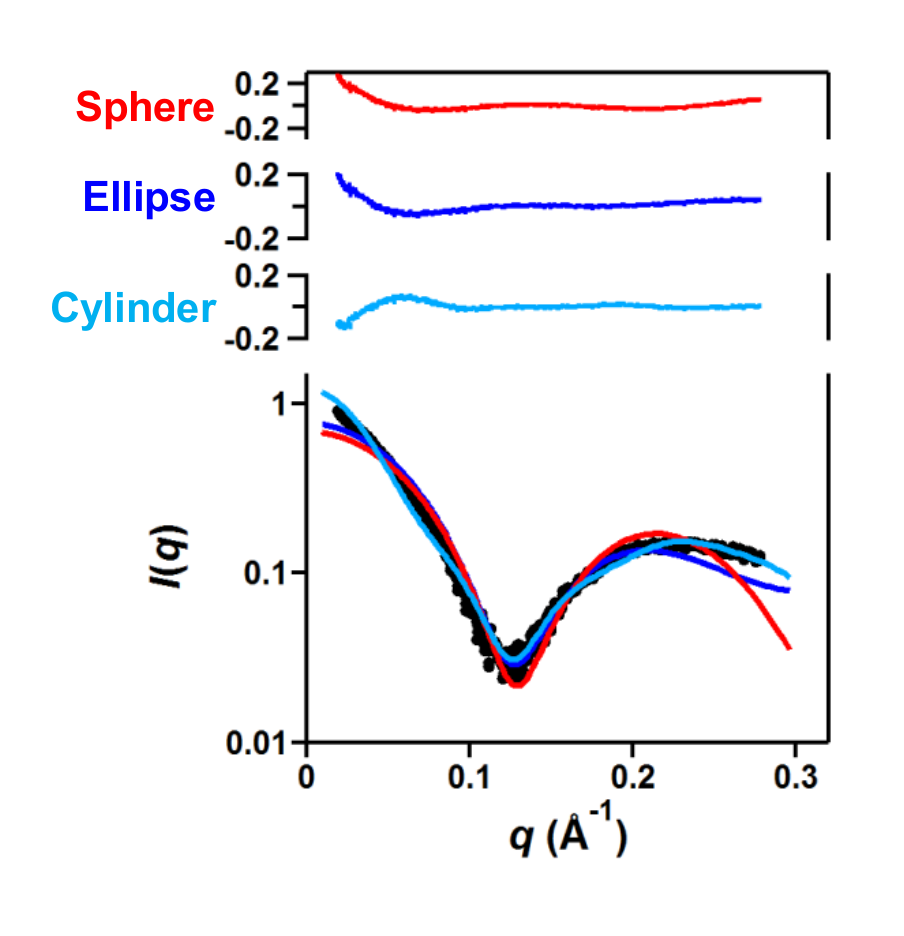

Supplement: Figure S10 — Comparison of sphere (red), oblate ellipse (blue), and cylinder (cyan) geometrical model fits to OG scattering data. The sphere model contains a core radius of 17.1 Å with a shell thickness of 3.6 Å. The ellipse model is oblate and contains a core with semi-axes of 21.4 Å and 10.5 Å with a shell thickness of 3.1 Å. The cylinder model has a core radius of 12.3 Å with a shell thickness of 2.9 Å, and total length of 92.9 Å. Core, shell, and solvent contrasts were consistent between the models. (TIF) [file pone.0062488.s010.tif]

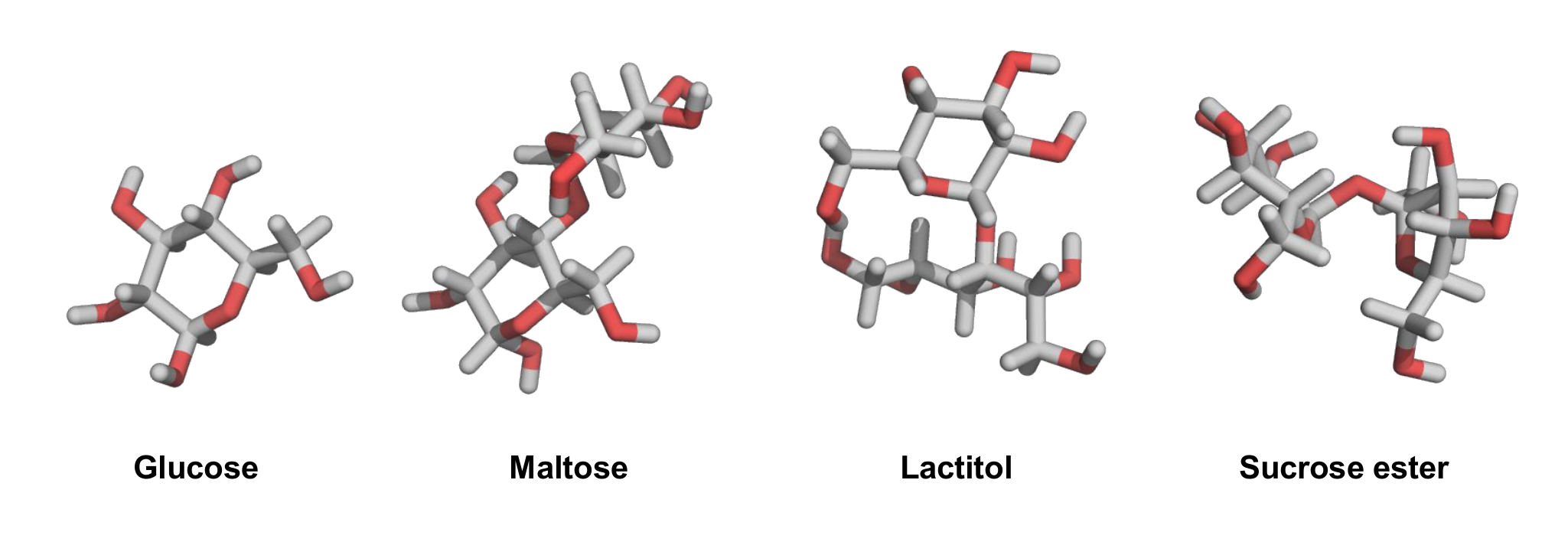

Supplement: Figure S11 — Head groups of each of the nonionic detergents compared in Figure 4. The atom attached to the alkyl chain is indicated by an arrow. The head groups are in order of increasing steric bulk from left to right (glucose<maltose<lactitol ≈ sucrose ester). (TIF) [file pone.0062488.s011.tif]
