# Supplementary material for: Dependence of Micelle Size and Shape on Detergent Alkyl Chain Length and Head Group
Source: PLoS One. 2013 May 8;8(5):e62488. doi: 10.1371/journal.pone.0062488 (PMC3648574; doi:10.1371/journal.pone.0062488)
Supplement: Table S1 — Values of geometric model fits to the scattering profiles for each detergent studied classified by model shape (oblate/prolate ellipsoid and sphere). (DOCX) [file pone.0062488.s012.docx]

**Table S1. Values of geometric model fits to the scattering profiles for each detergent studied classified by model shape (oblate/prolate ellipsoid and sphere).** Model fits are shown in Figure S4.

| Detergent | Oblate Ellipsoid | | | Prolate Ellipsoid | | | Sphere | |
| --- | --- | --- | --- | --- | --- | --- | --- | --- |
|  | a (Å) | b (Å) | t (Å) | a (Å) | b (Å) | t (Å) | r (Å) | t (Å) |
| FC10 | 11.0 | 18.8 | 2.7 | 20.4 | 13.4 | 2.8 | 15.2 | 2.7 |
| FC12 | 14.5 | 21.5 | 2.8 | 24.4 | 16.1 | 2.8 | 18.3 | 2.7 |
| FC14 | 16.5 | 25.0 | 2.8 | 29.7 | 18.8 | 2.8 | 21.1 | 2.7 |
| OM | 11.1 | 18.5 | 5.6 | 23.3 | 13.4 | 5.3 | 15.5 | 6.5 |
| DM | 13.5 | 22.8 | 5.5 | 27.4 | 16.5 | 5.4 | 19.0 | 6.4 |
| DDM | 15.7 | 28.0 | 5.6 | 32.0 | 19.5 | 5.4 | 21.9 | 5.9 |
| OG | 11.1 | 21.0 | 3.1 | 33.6 | 13.3 | 3.1 | 17.0 | 3.6 |
| NG | 12.0 | 24.6 | 3.2 | 45.0 | 14.4 | 3.2 | 18.3 | 3.4 |
| DG | 13.5 | 24.0 | 3.1 | 60.0 | 15.5 | 3.1 | 20.7 | 3.3 |
| LMPG | 17.1 | 24.0 | 5.6 | 28.5 | 18.7 | 5.5 | 21.0 | 6.0 |
| LPPG | 19.5 | 28.9 | 5.6 | 30.5 | 22.0 | 5.5 | 25.2 | 6.2 |

All values are reported in angstroms (Å) and have an error of ±0.5 Å in the axial dimensions, and ±0.3 Å in thickness. The values for the glucoside detergent fits are provided as estimates, as model fits (particularly NG and DG) contained deviations at low-*q*, and increased the uncertainty of the overall micelle length.
