# Supplementary material for: Dependence of Micelle Size and Shape on Detergent Alkyl Chain Length and Head Group
Source: PLoS One. 2013 May 8;8(5):e62488. doi: 10.1371/journal.pone.0062488 (PMC3648574; doi:10.1371/journal.pone.0062488)
Supplement: Methods S1 — Supporting information for methods of determining micelle size and shape from small angle X-ray scattering data. (DOCX) [file pone.0062488.s013.docx]

**Methods S1: Supporting information for methods of determining micelle size and shape from small angle X-ray scattering data.**

**Scattering theory for macromolecular size and shape determination.** In monodisperse solutions of globular particles, the measured scattering intensity as a function of momentum transfer *q* is given by

*I*(*q*) = *cP*(*q*)*S*(*q,c*) (1)

where *c* is the particle concentration, *P*(*q*) is the form (or particle structure) factor, and *S*(*q,c*) is the solution structure factor [1,2]. The form factor *P*(*q*) represents the scattering contributions from a single particle and can be directly calculated from the particle’s geometric shape. The solution structure factor *S*(*q,c*) accounts for interactions between particles in solution and modifies the measured scattering profile at finite concentrations [2-6]. The solution structure factor *S*(*q,c*) modifies *I*(*q*) most strongly at small momentum transfer *q*; however, for weakly interacting particles at dilute concentrations, *S*(*q,c*) is equal to unity and the scattering intensity is solely due to the particle form factor.

In the limit that interparticle correlations are negligible, the scattering intensity for very low momentum transfer *q* is given by the Guinier approximation [7,8]

$I(q)\approx I(0)e^{\frac{-Rg^{2}q^{2}}{3}}$ (2)

Thus, the forward scattering intensity *I*(0) and radius of gyration *Rg* can be determined by linearly fitting the low-*q* data in a plot of ln(*I*) as a function of *q*^2^. Linear fits were evaluated using the program PRIMUS [9] limiting the upper bound of the fitting range such that $q\cdot Rg\leq1.3$ [2]. A Guinier analysis of the experimental SAXS profiles at several detergent concentrations was performed to probe the effects of increasing concentration and to obtain reliable estimates of the micellar *Rg* and *I*(0). The radii of gyration (*Rg_expt_*) determined from Guinier fits to the low-*q* experimental data are presented in Table 2 for each detergent studied.

**Micelle size and shape determined from form factor model fits.** The X-ray scattering amplitude of a particle with electron density *ρ*(**r**) is given by

$A(q)=\int(\rho(r)-\rho_{s})e^{(iq\cdot r)}d^{3}r$ (3)

and the particle form factor is equal to the square of the amplitude, averaged over all orientations, $P(q)=\left\langle\left|A(q)\right|^{2}\right\rangle_{\Omega}$. To avoid effects of interparticle interference or detergent concentration on micelle size, scattering profiles at low detergent concentrations (typically ≤ 1 mM of micelle) were used for model fitting. A two-component ellipsoid model was employed to represent the detergent micelles, having an inner core and outer shell with different electron densities (*ρ_1_* and *ρ_2_*, respectively) and different dimensions. The electron densities can be estimated by dividing the number of electrons in either the tail or the head group by the volume of each of the chemical moieties [10]. The ellipsoid model contains a core having semi-axes *a* and *b* with an outer shell defined by the head group, having a uniform thickness *t* (Figure 1). Thus, the particle form factor for the ellipsoid micelle is given by

$P(q)=\int_{0}^{1} (3V_{1}(\rho_{1}-\rho_{2})\frac{j_{1}(u_{1})}{u_{1}}+3(V_{1}+V_{2})(\rho_{2}-\rho_{s})\frac{j_{1}(u_{2})}{u_{2}})^{2}$ (4)

Where , $u_{2}=q(a+t)^{2}x^{2}+(b+t)^{2}(1-x^{2}))^{\frac{1}{2}}$, the core volume $V_{1}=\frac{4}{3}\pi ab^{2}$, and the total volume $V_{1}+V_{2}=\frac{4}{3}\pi(a+t)(b+t)^{2}$. For a < b, the ellipsoid is oblate and, for a > b, it is prolate (Figure 1).

A nonlinear least-squares fitting routine implemented in Igor Pro (WaveMetrics) as part of the NCNR analysis toolkit [11] was used to fit two-component (core-shell) prolate and oblate ellipsoids (as well as spherical and cylindrical models) to the full scattering profiles and the best-fit model had the lowest residuals. Although designed for model fits to neutron scattering profiles, this procedure was readily adapted to produce form factors consistent with SAXS measurements by replacing the scattering length densities (based on nuclear interactions in SANS) with electron densities. If two models appear to fit the data equally well, potential steric violations in each model should be considered first. For example, a minor axis length corresponding to an alkyl chain length that is longer than the maximum possible extended chain length results in an invalid model. Additionally, the model-independent measurement of micelle parameters must agree with the model parameters to validate of the best model choice. Rather than adding complexity (*e.g.* a hydration layer or polydispersity) to improve model fits, the quality of model fits was sufficient to distinguish between model shapes and provide quantitative changes in micelle size.

The radius of gyration can also be calculated from the form factor model according to the relationship

$Rg^{2}=\frac{1}{5}\frac{(\rho_{2}-\rho_{s})(a+t)(b+t)^{2}[(a+t)^{2}+2(b+t)^{2}]+(\rho_{1}-\rho_{2})ab^{2}(a^{2}+2b^{2})}{(\rho_{2}-\rho_{s})(a+t)(b+t)^{2}+(\rho_{1}-\rho_{2})ab^{2}}$ (5)

where *ρ_1_*, *ρ_2_*, *ρ_s_*, *a*, *b*, and *t* are the aforementioned geometric model parameters. The calculated radius of gyration (*Rg_model_*) was compared to the radius of gyration from the Guinier analysis (*Rg_expt_*), which is determined in a model-independent fashion.

**Determination of aggregation numbers.** Aggregation numbers of micelles were estimated using two methods. The first approach considers that the scattering intensity of the micelle is directly proportional to the number *N* detergent monomers in the micelle. In the absence of interparticle interference effects, the forward scattering intensity is proportional to the square of the total scattering contrast [2,7]

$I(0)=\kappa c[V(\rho-\rho_{s})]^{2}$ (6)

The total scattering contrast is $V(\rho-\rho_{s})$, where *V* is the molecular volume, *ρ* is the average electron density of the particle, and *ρ*_s_ is the electron density of the solvent. The calculated electron density (*ρ*_s_) of the buffer used (20 mM phosphate buffer, pH 6.2, 150 mM NaCl, and 10% by vol. D_2_O) is 0.337 – 0.340 e/Å^3^ [12]. *κ* is a proportionality constant that is determined from the measurements of molecular weight standards (see Sample preparation section) of known concentration, molecular volume, and electron density. To represent the concentration of micelles, the total detergent concentration is adjusted by the cmc (*c* – cmc). The micelle aggregation number can then be calculated by comparing the measured *I*(0)_det_ (from Guinier analysis) to that expected for the detergent monomer [10]:

$N_{\exp t}=\frac{I(0)_{\det}}{I(0)_{mon}}=\frac{I(0)_{\det}}{\kappa(c-cmc)(\rho_{\det}-\rho_{s})^{2}V_{mon}^{2}}$ (7)

The detergent monomer electron densities *ρ_det_* were calculated by dividing the number of electrons by the molecular volume *V_mon_*. The detergent monomer volumes were calculated from specific densities [13] using the Tanford formula [14] (*V_tail_* = 27.4 + 26.9 · *n_c_*) to adjust for different chain lengths.

For the second approach, aggregation numbers were calculated from the dimensions of the form factor core-shell models. The total volume of the hydrophobic core was divided by the alkyl chain (tail) volume per monomer

$N_{\mathrm{mod}el}=\frac{\frac{4}{3}\pi ab^{2}}{V_{tail}}$ (8)

to obtain the aggregation number *N_model_* of the micelle.

**Model independent assessment of micelle dimensions.** The investigated detergent micelles exhibit a characteristic second maximum in the SAXS profile, arising from the large difference in scattering contrast between the core and shell. The position of this second maximum is independent of detergent concentration and is denoted by *q_max_*. The corresponding length scale in real space is 2*π*/*q_max_*. This real space distance has been shown to correlate well with the maximal thickness across the alkyl chain core along the short dimension of the micelle, or the dominant head group – head group distance *L_expt_* [10]. This distance can be approximated from the model as *L_model_* = 2*a* + *t* for oblate and *L_model_* = 2*b* + *t* for prolate ellipsoids defined by semi-axis *a*, semi-axis *b*, and an outer shell of thickness *t* (Figure 1).

**References**

1. Chen S (1986) Small angle neutron scattering studies of the structure and interaction in micellar and microemulsion systems. Annu Rev Phys Chem 37: 351-399.

Available: http://www.annualreviews.org/doi/abs/10.1146/annurev.pc.37.100186.002031.

2. Svergun DI, Koch M (2003) Small-angle scattering studies of biological macromolecules in solution. Rep Prog Phys 66: 1735-1782.

Available: http://iopscience.iop.org/0034-4885/66/10/R05.

3. Bendedouch D, Chen S, Koehler WC (1983) Determination of interparticle structure factors in ionic micellar solutions by small angle neutron scattering. J Phys Chem 87: 2621-2628. Available: http://pubs.acs.org/doi/abs/10.1021/j100237a030.

4. Hayter JB, Penfold J (1981) An analytic structure factor for macroion solutions. Mol Phys 42: 109-118. Available: http://www.tandfonline.com/doi/abs/10.1080/00268978100100091.

5. Kotlarchyk M, Chen S (1983) Analysis of small angle neutron scattering spectra from polydisperse interacting colloids. J Chem Phys 79: 2461-2469.

Available: http://jcp.aip.org/resource/1/jcpsa6/v79/i5/p2461_s1.

6. Hayter JB (1985). Proc Int Sch Phys “Enrico Fermi” 90: 59-92.

7. Glatter O (1982) Small angle x-ray scattering. London, UK: Academic Press.

8. Guinier, A (1939). Ann. Phys. (Paris) 12: 161-237.

9. Konarev P, Volkov V, Sokolova A, Koch M, Svergun D (2003) PRIMUS: a Windows PC-based system for small-angle scattering data analysis. J Appl Cryst 36: 1277-1282.

Available: http://scripts.iucr.org/cgi-bin/paper?ks5004.

10. Lipfert J, Columbus L, Chu VB, Lesley SA, Doniach S (2007) Size and shape of detergent micelles determined by small-angle X-ray scattering. J Phys Chem B 111: 12427-12438. Available: http://pubs.acs.org/doi/abs/10.1021/jp073016l.

11. Kline S (2006) Reduction and analysis of SANS and USANS data using IGOR Pro. J Appl Cryst 39: 895-900. Available: http://scripts.iucr.org/cgi-bin/paper?do5025.

12. Schiel J, Hage DS (2005) Density measurements of potassium phosphate buffer from 4 to 45° C. Talanta 65: 495-500.

Available: http://www.sciencedirect.com/science/article/pii/S003991400400373X.

13. Le Maire M, Champeil P, Moller JV (2000) Interaction of membrane proteins and lipids with solubilizing detergents. Biochim Biophys Acta 1508: 86-111.

Available: http://www.sciencedirect.com/science/article/pii/S0304415700000101.

14. Tanford C (1980) The hydrophobic effect: formation of micelles and biological membranes. New York: Wiley.
