# Supplementary material for: Dependence of Micelle Size and Shape on Detergent Alkyl Chain Length and Head Group
Source: PLoS One. 2013 May 8;8(5):e62488. doi: 10.1371/journal.pone.0062488 (PMC3648574; doi:10.1371/journal.pone.0062488)
Supplement: Results S1 — Supporting information and results of micelle physical properties with comparison to relevant studies. (DOCX) [file pone.0062488.s014.docx]

**Results S1: Supporting information and discussion of the sizes, shapes, and physical properties of the investigated detergent micelles.**

**Phosphocholines.** Phosphocholine detergents have zwitterionic head groups similar to those of phosphocholine lipids found in biological membranes. Alkyl phosphocholines (specifically FC12) have facilitated solution NMR membrane protein structure determination


[1] and maintain functional activity of some membrane-bound enzymes


[2,3]. Despite the common use of FC12, the structure of the pure micelle and detergent organization in PDCs are not agreed upon in the literature. A molecular modeling study of FC12 indicated that a spherical micelle shape was preferred when aggregation numbers of 54 or 65 were used; however, a prolate ellipsoid geometry was preferred when only 40 monomers per micelle were used


[4]. The recent SAXS-based investigation of phosphocholine micelles, with and without peptide components, by Göbl *et al*. used a spherical approximation for all micelles and PDCs


[5]. In contrast, other SAXS measurements reported improved model fits using prolate ellipsoid micelle shapes, rather than spherical, for FC10 and FC12


[6].

The scattering profiles of the zwitterionic phosphocholine detergents FC10, FC12, and FC14, with alkyl chain lengths of *n* = 10, 12, and 14, respectively, were best fit with prolate core-shell ellipsoids. A comparison of different model fits to the experimental data for each alkyl chain length of phosphocholine detergents at ~1 mM micelle concentration (a typical concentration used in NMR studies) are presented in Figure S9A, with the associated model fit parameters listed in Tables 2 and S1. FC10 and FC12 were independently investigated in this study and agreed with the previously published data


[6] (Figures S2-S3). FC14 was previously uncharacterized; therefore, the concentration series of scattering profiles, Guinier plots, calculated aggregation numbers, and the best model fit to the lowest detergent concentration (to reduce contributions from interparticle interference) are provided in Figure 2. The phosphocholine micelle models share a common 2.7-3.0 Å uniform shell thickness, a prolate ellipsoid geometry, and a core minor axis approximating the length of a detergent monomer with almost fully extended alkyl chains.

The investigation by Göbl *et al.* reported spherical model fits with radii of 22.5 Å (FC10), 27.0 Å (FC12), and 32.0 Å (FC14), including a 10.4-11.4 Å head group shell thickness


[5]. This spherical model presents a radius that is much larger (by about 8-10 Å) than the predicted maximum extension of the alkyl chain tail. With the restriction in the packing of the detergent that has the head groups exposed to the aqueous solvent, this model would require additional volume in the center of the micelle not accounted for by the detergent atoms. Despite the physical unlikelihood of these spherical models, spherical model fits were optimized to the phosphocoline SAXS scattering profiles (Figure S9A, right), but resulted in the largest deviations from the experimental data when compared to prolate and oblate ellipsoids (Figure S9A, left and center).

**Maltosides.** The nonionic maltoside detergents have an uncharged disaccharide (maltose) head group with a single alkyl chain tail. Maltoside detergents (particularly DM and DDM) are extensively used in the preparation (extraction, solubilization, and purification)


[7-9] and crystallization of membrane proteins


[10]. About half of the high-resolution X-ray crystal structures of α-helical membrane proteins were determined using maltoside detergents


[11,12]. Additionally, DDM was used to study the unfolding of *E.coli* DsbB,


[13] and to study conformational changes in mammalian rhodopsin by ^19^F NMR


[14]. Although the maltoside detergents are widely used in membrane protein studies, maltoside detergent micelles have not previously been systematically studied. In addition, as with the phosphocholines, there are conflicting reports in the literature on the shapes and sizes of maltoside micelles


[6,15,16].

The scattering profiles of the maltoside detergents OM, DM, and DDM, containing alkyl chain lengths of *n* = 8, 10, and 12, respectively, were best fit with oblate core-shell ellipsoid models. A comparison of different models fit to the experimental data for each alkyl chain length of maltoside detergents at a micelle concentration of ~1 mM is presented in Figure S9B, with the associated fit parameters given in Table 2 and S1. Scattering data for OM was not previously published; therefore, the concentrations series scattering profiles, Guinier plots, calculated aggregation numbers, and the best model fit to the lowest detergent concentration (to reduce contributions from interparticle interference) for OM are provided in Figure 2. DM and DDM were independently investigated in this study (Figures S4-S5) and similar to previously published data


[6]. The maltoside micelle models share a common 5.4-5.8 Å uniform shell thickness, an oblate ellipsoid geometry, and a minor core axis approximating the length of a detergent monomer with almost fully extended alkyl chains. As with the phosphocholines, the spherical model exhibited the largest deviation from the experimental data (Figure S9B, right). The oblate and prolate model fits were of similar quality (Figure S9B, left and center); however, the prolate (as well as the sphere) fit parameters were physically unreasonable because the short axis was greater than the maximum extended alkyl chain length (Table S1). The oblate geometry, with the short axis consistent with the chain length, was also preferred because of the agreement between the measured and calculated *L* and *Rg* values (Table 2).

Using an approach similar to that of Göbl *et al.*


[5], He *et al.* reported that OM (in water) formed spherical micelles with an aggregation number of 26, hydrophobic core radius of 11.5 Å, and a total micelle radius of 23.7 Å


[16]. Although the spherical core radius is comparable to the length of the extended alkyl chain and minor elliptical axis presented here, the shell thickness (12.2 Å) is approximately twice that of the observed shell thickness in this study. In addition, the proposed 12.2 Å head group shell is longer that the alkyl chain, which is not expected based on the monomeric structure. In contrast, Dupuy *et al.* determined DDM (in water) to be an oblate ellipsoid with a major hydrophobic core radius of 28.2 Å, shell thickness of 6.2 Å, and ellipticity of 0.50 – 0.60


[15], in very close agreement with our results (Table 2).

**Glucosides.** The nonionic glucoside detergents have a monosaccharide (glucose) head group with a single alkyl chain tail. Short-chain alkyl glucosides (specifically OG) have been successful in the extraction, purification, and crystallization of many membrane proteins [17,18]. Micelles formed by glucoside detergents have been reported to be polydisperse, have concentration dependent aggregation, and interparticle repulsion


[19-21]. The proposed models for glucoside micelles vary in shape (sphere


[22], oblate


[6], and cylinder


[23]) and size (e.g., large range aggregation numbers reported


[19,24]). In order to elucidate the size and shape of micelles formed by glucoside detergents, we investigated three glucosides varying in alkyl chain length.

The scattering profiles of the nonionic glucoside detergents OG, NG, and DG, containing alkyl chain lengths of *n* = 8, 9, and 10, respectively, were fit with an oblate core-shell ellipsoid model. A comparison of different models fit to the experimental data for each alkyl chain length of glucoside detergent at ~50 mM total detergent monomer concentration is presented in Figure S9C, with the corresponding model fit parameters given in Tables 2 and S1. The glucoside detergent parameters are presented at a total detergent monomer concentration of 50 mM due to solubility constraints and phase behavior of NG and DG. The corresponding micelle concentration is difficult to determine for OG and DG due to the wide range of aggregation numbers reported (Table 1). The concentrations series scattering profiles, Guinier plots, and the best model fit to the lowest detergent concentration (to reduce contributions from interparticle interference) for DG are provided in Figure 2. OG and NG were independently investigated in this study (Figures S6-S7) with results similar to previously published data


[6]. The glucoside micelle models share a common 2.9-3.5 Å uniform shell thickness, and an oblate ellipsoid geometry with the minor axis approximating the length of a detergent monomer with almost fully extended alkyl chains. The oblate and prolate model fits were of similar quality (Figure S9C); however, the prolate (as well as the sphere) fit parameters were physically unreasonable because the short axis was greater than the maximum extended alkyl chain length (Table S1). Because of the conflicting observations reported in the literature, core-shell cylindrical models were also investigated, but did not produce more reasonable fits (Figure S10).

The sharp increase in glucoside scattered intensity at low *q* values may indicate polydispersity of the size distribution or the presence of larger aggregate structures (Figure 2). When the surface area of a globular micelle equals that of a cylindrical micelle, a transition zone with a bimodal distribution of spherical and cylindrical micelles might be expected


[25] and could account for the observed scattering profile. The effects at low *q* presented inherent difficulties on the Guinier analysis and model fitting, so physical parameters such as aggregation number and radii of gyration were approximated (Table 2). Unfortunately, the lack of a reliable extrapolation of the glucoside scattering data to zero scattering angle (*I(0)*) precluded calculation of the corresponding aggregation number.

Zhang *et al.* simultaneous fit X-ray and neutron scattering data over a very broad *q*-range (0.008 – 0.8 Å^-1^) with elongated two-component cylindrical models for heptyl-glucoside (HG), OG, and NG micelles (in water)


[26]. The resulting model of OG micelles (~1 mM) yielded an aggregation number of 49-53, inner radius of 10.6 Å, shell thickness of 2.5 Å, and length of 34.8 Å. The model of NG (~0.25 mM) micelles yielded an aggregation number of 1800-3600, inner radius of 12.1 Å, shell thickness of 2.4 Å, and length of 1100-2100 Å. He *et al*. also fit SANS data for OG using both a cylindrical model having an aggregation number of 90, hydrocarbon core radius of 8.5 Å and overall length of 88 Å with a shell thickness of 4.2 Å along the center of the rod and 4 Å at each end, as well as, a prolate ellipsoidal model having a major axis length of 63.2 Å and minor axis length of 12.9 Å


[27]. The shell thickness in these studies and that reported here are similar with Zhang *et al.* (~0.5 Å thinner) and that of He *et al.* (~0.5 Å thicker). With respect to the core dimensions of the cylindrical models presented by Zhang *et al.*, the values corresponding to extended alkyl chain lengths are the similar to those presented here. However, the cylindrical model of OG by He *et al.* has a shorter dimension (by ~2 Å), while the prolate model from the same data is longer (by ~2 Å). The aggregation numbers from the cylindrical models are quite large by comparison, and have a significant impact when determining micelle concentration. Tanford proposes that cylindrical micelles can grow in length (as concentration increases) without limit because the surface area per headgroup is independent of the length of the cylinder


[25]. Therefore, the discrepancy between the published models of the glucoside micelles is likely due to a polydisperse mixture of cylindrical and ellipsoid micelles the ratio of which is highly concentration dependent.

**Lysophosphatidyl glycerols.** Lysophosphatidyl glycerol (LPG) detergents contain a negatively charged phosphate in the head group resulting in a micelle with negative surface charge. LPG detergents have head groups similar to biological phosphatidyl glycerol lipids, but lack a second alkyl chain. LMPG (n=14) and LPPG (n=16) are emerging as useful detergents in membrane protein structural


[28,29] and functional


[30] investigations. However, recombinant human KCNE1, a bitopic modulator of the voltage-gated potassium channel KCNQ1, is the only membrane protein structure in LMPG micelles reported to date


[31]. Despite promising applications of LPG detergents in membrane protein studies and the potential advantages afforded by modulating micelle surface charge through ionic detergents, a thorough characterization of the LPG micelle size and shape at varied alkyl chain lengths has not previously been reported.

The scattering profiles of the ionic LPG detergents, LMPG and LPPG were best fit with an oblate core-shell ellipsoid model. A comparison of model fits to the experimental data for each alkyl chain length of LPG are presented in Figure S9D, at total detergent concentrations of 16 mM for LMPG and 25 mM for LPPG, with the associated model fit parameters listed in Table 2 and S1. The physical parameters for LPPG were previously determined (Figure S8). The concentrations series scattering profiles, Guinier plots, calculated aggregation numbers, and the best model fit to the lowest detergent concentration (to reduce contributions from interparticle interference) for LMPG are presented in Figure 2. The LPG micelle models share a common 5.3-6.1 Å uniform shell thickness, and an oblate ellipsoid geometry with the minor axis approximating the length of a detergent monomer with almost fully extended alkyl chains. The prolate and sphere model fits are of lesser quality as assessed by the residuals and are less plausible because the short axis was greater than the maximum extended alkyl chain length (Table S1).

Strong interparticle interference is observed in LPG micelles (most notable at high detergent concentrations), likely due to electrostatic repulsions between the negatively-charged micelles. Evidence for this interaction is provided by the observation of a decreasing slope (and thus lower *I*(*0*) and *Rg*) in the Guinier region at low scattering angles with increased detergent concentration (Figure 2B). Therefore, the parameters determined from the Guinier analysis and forward scattering for these detergents underestimate their actual values. In order to minimize these effects, the low concentration data were used for analysis whenever possible.

**References**

1. Qureshi T, Goto NK (2012) Contemporary methods in structure determination of membrane proteins by solution NMR. Top Curr Chem 326: 123-185.

Available: http://www.springerlink.com/content/48501481216310u5/.

2. Czerski L, Sanders CR (2000) Functionality of a membrane protein in bicelles. Anal Biochem 284: 327-333. Available: http://www.sciencedirect.com/science/article/pii/S0003269700947201.

3. Vinogradova O, Sönnichsen F, Sanders CR (1998) On choosing a detergent for solution NMR studies of membrane proteins. J Biomol NMR 11: 381-386.

Available: http://www.springerlink.com/content/k88741378lv68804/.

4. Tieleman DP, van der Spoel D, Berendsen H (2000) Molecular dynamics simulations of dodecylphosphocholine micelles at three different aggregate sizes: micellar structure and chain relaxation. J Phys Chem B 104: 6380-6388.

Available: http://pubs.acs.org/doi/abs/10.1021/jp001268f.

5. Gobl C, Dulle M, Hohlweg W, Grossauer J, Falsone SF, et al. (2010) Influence of phosphocholine alkyl chain length on peptide-micelle interactions and micellar size and shape. J Phys Chem B 114: 4717-4724. Available: http://pubs.acs.org/doi/abs/10.1021/jp9114089.

6. Lipfert J, Columbus L, Chu VB, Lesley SA, Doniach S (2007) Size and shape of detergent micelles determined by small-angle X-ray scattering. J Phys Chem B 111: 12427-12438. Available: http://pubs.acs.org/doi/abs/10.1021/jp073016l.

7. Arnold T, Linke D (2008) The use of detergents to purify membrane proteins. Curr Prot Protein Sci 53: 4.8.1-4.8.30.

Available: http://onlinelibrary.wiley.com/doi/10.1002/0471140864.ps0408s53/full.

8. Ruan K, Wu J, Cervantes V (2008) Characterization of the substrate mimic bound to engineered prostacyclin synthase in solution using high-resolution NMR spectroscopy and mutagenesis: implication of the molecular mechanism in biosynthesis of prostacyclin. Biochemistry 47: 680-688. Available: http://pubs.acs.org/doi/abs/10.1021/bi701671q.

9. Wetterholm A, Molina DM, Nordlund P, Eshaghi S, Haeggstrom JZ (2008) High-level expression, purification, and crystallization of recombinant rat leukotriene C(4) synthase from the yeast Pichia pastoris. Protein Expression Purif 60: 1-6.

Available: http://www.sciencedirect.com/science/article/pii/S1046592808000697.

10. Privé GG (2007) Detergents for the stabilization and crystallization of membrane proteins. Methods 41: 388-397.

Available: http://www.sciencedirect.com/science/article/pii/S1046202307000102.

11. Hong W, Baker KA, Ma X, Stevens RC, Yeager M, et al. (2010) Design, synthesis, and properties of branch-chained maltoside detergents for stabilization and crystallization of integral membrane proteins: human connexin 26. Langmuir 26: 1-7.

Available: http://pubs.acs.org/doi/abs/10.1021/la904893d.

12. de Grip WJ (1982) Thermal stability of rhodopsin and opsin in some novel detergents. Methods Enzymol 81: 256-265.

Available: http://www.sciencedirect.com/science/article/pii/S0076687982810409.

13. Sehgal P, Otzen DE (2009) Thermodynamics of unfolding of an integral membrane protein in mixed micelles. Protein Sci 15: 890-899.

Available: http://onlinelibrary.wiley.com/doi/10.1110/ps.052031306/full.

14. Klein-Seetharaman J, Getmanova EV, Loewen MC, Reeves PJ, Khorana HG (1999) NMR spectroscopy in studies of light-induced structural changes in mammalian rhodopsin: applicability of solution (19)F NMR. Proc Natl Acad Sci 96: 13744-9.

Available: http://www.pnas.org/content/96/24/13744.short.

15. Dupuy C, Auvray X, Petipas C, Rico-Lattes I (1997) Anomeric effects on the structure of micelles of alkyl maltosides in water. Langmuir 13: 3965-3967.

Available: http://pubs.acs.org/doi/abs/10.1021/la9604285.

16. He L, Garamus V, Funari S, Malfois M, Willumeit R, et al. (2002) Comparison of small-angle scattering methods for the structural analysis of octyl-β-maltopyranoside micelles. J Phys Chem B 106: 7596-7604. Available: http://pubs.acs.org/doi/abs/10.1021/jp020034o.

17. Hite RK, Li Z, Walz T (2010) Principles of membrane protein interactions with annular lipids deduced from aquaporin-0 2D crystals. EMBO J 29: 1652-1658.

Available: http://www.nature.com/doifinder/10.1038/emboj.2010.68.

18. Lu W, Wacker T, Gerbig-Smentek E, Andrade SL, Einsle O (2011) pH-Dependent Gating in a FocA Formate Channel. Science 332: 352-354.

Available: http://www.sciencemag.org/cgi/doi/10.1126/science.1199098.

19. Nilsson F, Soderman O, Hansson P, Johansson I (1998) Physical−Chemical Properties of C9G1 and C10G1 β-Alkylglucosides. Phase Diagrams and Aggregate Size/Structure. Langmuir 14: 4050-4058. Available: http://pubs.acs.org/doi/abs/10.1021/la980261a.

20. Cohen DE, Thurston GM, Chamberlin RA, Benedek GB, Carey MC (1998) Laser Light Scattering Evidence for a Common Wormlike Growth Structure of Mixed Micelles in Bile Salt− and Straight-Chain Detergent−Phosphatidylcholine Aqueous Systems:  Relevance to the Micellar Structure of Bile. Biochemistry 37: 14798-14814.

Available: http://pubs.acs.org/doi/abs/10.1021/bi980182y.

21. Persson CM, Claesson PM, Johansson I (2000) Interfacial Behavior of n-Octyl β- -Glucopyranoside Compared to That of a Technical Mixture Consisting of Octyl Glucosides. Langmuir 16: 10227-10235. Available: http://pubs.acs.org/doi/abs/10.1021/la000665e.

22. Thiyagarajan P, Tiede DM (1994) Detergent micelle structure and micelle-micelle interactions determined by small-angle neutron scattering under solution conditions used for membrane protein crystallization. J Phys Chem 98: 10343-10351.

Available: http://pubs.acs.org/doi/abs/10.1021/j100091a058.

23. Giordano R, Maisano G, Teixeira J (1997) SANS Studies of Octyl-β-glucoside and Glycine Micellar Solutions. J Appl Cryst 30: 761-764. Available: http://scripts.iucr.org/cgi-bin/paper?S0021889897001817.

24. Lorber B, Bishop JB, DeLucas L (1990) Purification of octyl β-d-glucopyranoside and re-estimation of its micellar size. Biochim Biophys Acta, Biomembr. 1023: 254-265.

Available: http://linkinghub.elsevier.com/retrieve/pii/000527369090421J.

25. Tanford C (1980) The hydrophobic effect: formation of micelles and biological membranes. New York: Wiley.

26. Zhang R, Marone PA, Thiyagarajan P, Tiede DM (1999) Structure and Molecular Fluctuations of n-Alkyl-β-D-glucopyranoside Micelles Determined by X-ray and Neutron Scattering. Langmuir 15: 7510-7519. Available: http://pubs.acs.org/doi/abs/10.1021/la990076c.

27. He LZ, Garamus V, Niemeyer B, Helmholz H, Willumeit R (2000) Determination of micelle structure of octyl-β-glucoside in aqueous solution by small angel neutron scattering and geometric analysis. J Mol Liq 89: 239-249.

Available: http://linkinghub.elsevier.com/retrieve/pii/S0167732200900175.

28. Williams KA, Farrow NA, Deber CM, Kay LE (1996) Structure and Dynamics of Bacteriophage IKe Major Coat Protein in MPG Micelles by Solution NMR. Biochemistry 35: 5145-5157. Available: http://pubs.acs.org/doi/abs/10.1021/bi952897w.

29. Koehler J, Sulistijo ES, Sakakura M, Kim HJ, Ellis CD, et al. (2010) Lysophospholipid Micelles Sustain the Stability and Catalytic Activity of Diacylglycerol Kinase in the Absence of Lipids. Biochemistry 49: 7089-7099. Available: http://pubs.acs.org/doi/abs/10.1021/bi100575s.

30. Dufour JP, Goffeau A (1978) Solubilization by lysolecithin and purification of the plasma membrane ATPase of the yeast Schizosaccharomyces pombe. J Biol Chem 253: 7026-7032. Available: http://www.jbc.org/content/253/19/7026.short.

31. Kang C, Tian C, Sönnichsen FD, Smith JA, Meiler J, et al. (2008) Structure of KCNE1 and Implications for How It Modulates the KCNQ1 Potassium Channel. Biochemistry 47: 7999-8006. Available: http://pubs.acs.org/doi/abs/10.1021/bi800875q.
